# NOX2 Contributes to High‐Frequency Outer Hair Cell Vulnerability in the Cochlea

**DOI:** 10.1002/advs.202408830

**Published:** 2025-06-26

**Authors:** Meihao Qi, Zejun Gao, Yang Qiu, Renfeng Wang, Keyong Tian, Bo Yue, Xinyu Zhang, Peng Zhang, Ziqi Wu, Qingwen Zhu, Zhenzhen Liu, Zhuoyao Ma, Xueying Zhou, Yu Han, Jun Chen, Jianhua Qiu, DingJun Zha

**Affiliations:** ^1^ Department of Otolaryngology Head and Neck Surgery Xijing Hospital Air Force Medical University Xi'an 710032 China; ^2^ Department of Ophthalmology Hainan Hospital People's Liberation Army (PLA) General Hospital Sanya 572013 China; ^3^ Department of Ultrasound Diagnostics Tangdu Hospital Air Force Medical University Xi'an 710038 China

**Keywords:** high‐frequency vulnerability, NOX2, Nrf2, OHCs, ROS, single‐cell RNA sequencing

## Abstract

Most patients with sensorineural hearing loss (SNHL) may initially experience high‐frequency hearing loss linked to greater vulnerability of outer hair cells (OHCs) in the cochlear basal turn. However, the molecular mechanism underlying this susceptibility in the high‐frequency region remains unclear. Here, NOX2 is first identified as a differentially expressed gene related to oxidative damage in the apical and basal turns through single‐cell RNA sequencing. In mouse cochleae damaged by neomycin and noise, Nox2‐positive OHCs increase progressively from the apex to base, indicating that NOX2 expression clearly differed significantly between the apical and basal OHCs. In NOX2 knockout mice, OHC damage caused by neomycin and noise exposure is significantly reduced. Furthermore, knocking out NOX2 increases the expression of Nrf2 in the cytoplasm and Nrf2 nuclear translocation in OHCs. In addition, Nrf2 inhibitors counteract the protective effect of NOX2 knockout against neomycin. Finally, when Nox2 is used as the target, ginsenoside Rg1 is identified as a compound with protective effects against neomycin‐induced hearing loss. In summary, these results indicate NOX2 is highly correlated with high‐frequency OHCs vulnerability in the cochlea and is a potential intervention target for the clinical treatment of SNHL.

## Graphical abstract

This study first identifies NOX2 as a differentially expressed gene related to oxidative damage in the apical and basal turns through single‐cell RNA sequencing. NOX2 gene knockout mitigates OHCs damage caused by neomycin and noise and enhances Nrf2 expression and nuclear translocation. Targeting Nox2 protects against hearing loss, making Nox2 a potential therapeutic target for SNHL.

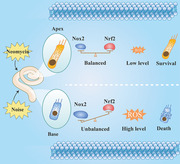

## 1 Introduction

Sensorineural hearing loss (SNHL) is a common disabling disease that severely affects oral communication and imposes a heavy economic and social burden globally. Most patients with SNHL likely originate from initial damage to the high‐frequency regions associated with the cochlear base.^[^ ^1^, ^2^ ^]^ The three rows of outer hair cells (OHCs) of the cochlea are arranged on a spiral‐shaped basilar membrane. The OHCs at the base of the cochlea sense high‐frequency sounds, whereas the OHCs at the apex of the cochlea sense low‐frequency sounds. High‐frequency hearing loss pathologically corresponds to damage to the basal OHCs of the cochlea.^[^ ^3^, ^4^ ^]^ After rats or mice are exposed to ototoxic drugs or noise, high‐frequency region damage occurs first, and the degree of damage to cochlear OHCs gradually increases from the apex to the base.^[^ ^5^ ^]^ The results mentioned above indicate that basal OHCs are more susceptible to damage than apical OHCs are; however, the specific mechanism has not yet been elucidated.

Previous studies have shown that ototoxic drugs, noise stimulation, aging, and other pathogenic factors can increase the level of reactive oxygen species (ROS) in the cochlea, leading to damage to and death of cochlear OHCs.^[^ ^6^, ^7^, ^8^, ^9^, ^10^ ^]^ Su‐Hua Sha reported that apical OHCs have more reduced glutathione than basal OHCs do and concluded that the vulnerability of basal OHCs to damage may be due to their lower tolerance to ROS than apical OHCs do.^[^ ^11^, ^12^ ^]^ In addition, the different uptake capacities of apical and basal OHCs for ototoxic drugs can also partially explain the difference in vulnerability to aminoglycoside ototoxicity.^[^ ^13^ ^]^

Apical and basal OHCs are located at various parts of the basilar membrane. In terms of the morphology of the cells, apex OHCs are longer than base OHCs. In terms of cell function, apical OHCs sense low‐frequency sounds, whereas basal OHCs sense high‐frequency sounds. From the perspective of pathology, basal OHCs are more susceptible to damage than apical OHCs are. Although apical and basal OHCs are essentially the same cell type, there are indeed differences in the biological behaviors of apical and basal OHCs, and we speculate that there are differences in their transcription levels. To date, no genes have been reported to be associated with the differential vulnerability of apical and basal hair cells. Therefore, clarifying the differences in gene expression levels between apical and basal OHCs is highly important for revealing the molecular mechanism of high‐frequency regions vulnerability in the cochlea.

The number of auditory hair cells in humans and mammals is only a few thousand to tens of thousands, and the number of hair cells that can be isolated for transcriptome sequencing is even more limited. The cochleae of rats or mice ossify when they grow, which makes it difficult to anatomically separate hair cells, especially basal OHCs. Therefore, it is challenging to study the differences between apical and basal OHCs.^[^ ^14^ ^]^ The emergence of single‐cell RNA sequencing (scRNA‐seq) technology in recent years has brought new opportunities to study the differences in the vulnerability of cochlear OHCs. ScRNA‐seq requires a sample size of one or dozens of cells and is suitable for the study of OHCs in different regions.^[^ ^15^ ^]^

In this study, we analyzed the transcriptome differences between apical and basal OHCs via scRNA‐seq and screened for genes that were differentially expressed in apical and basal OHCs. The relationship between the redox‐related differentially expressed gene NADPH oxidase 2 (NOX2) and high‐frequency cochlear vulnerability was studied in‐depth. In both neomycin‐induced and noise‐induced injury models, NOX2 expression significantly differed in apical and basal OHCs. We further investigated the key roles of Nox2 and Nrf2 (NF‐E2‐related factor 2) in the balance of oxidation and antioxidant activity in cochlear OHCs. Finally, when Nox2 was used as the target, ginsenoside Rg1 was identified as a compound with protective effects against neomycin‐induced hearing loss. In conclusion, the results of our study suggest that NOX2 is associated with differential vulnerability of apical and basal OHCs and may be a potential therapeutic target for intervention in hair cell damage and hearing loss caused by aminoglycoside antibiotics or noise.

## 2 Results

### 2.1 Isolation and Capture of Apical and Basal OHCs

**Figure 1:**
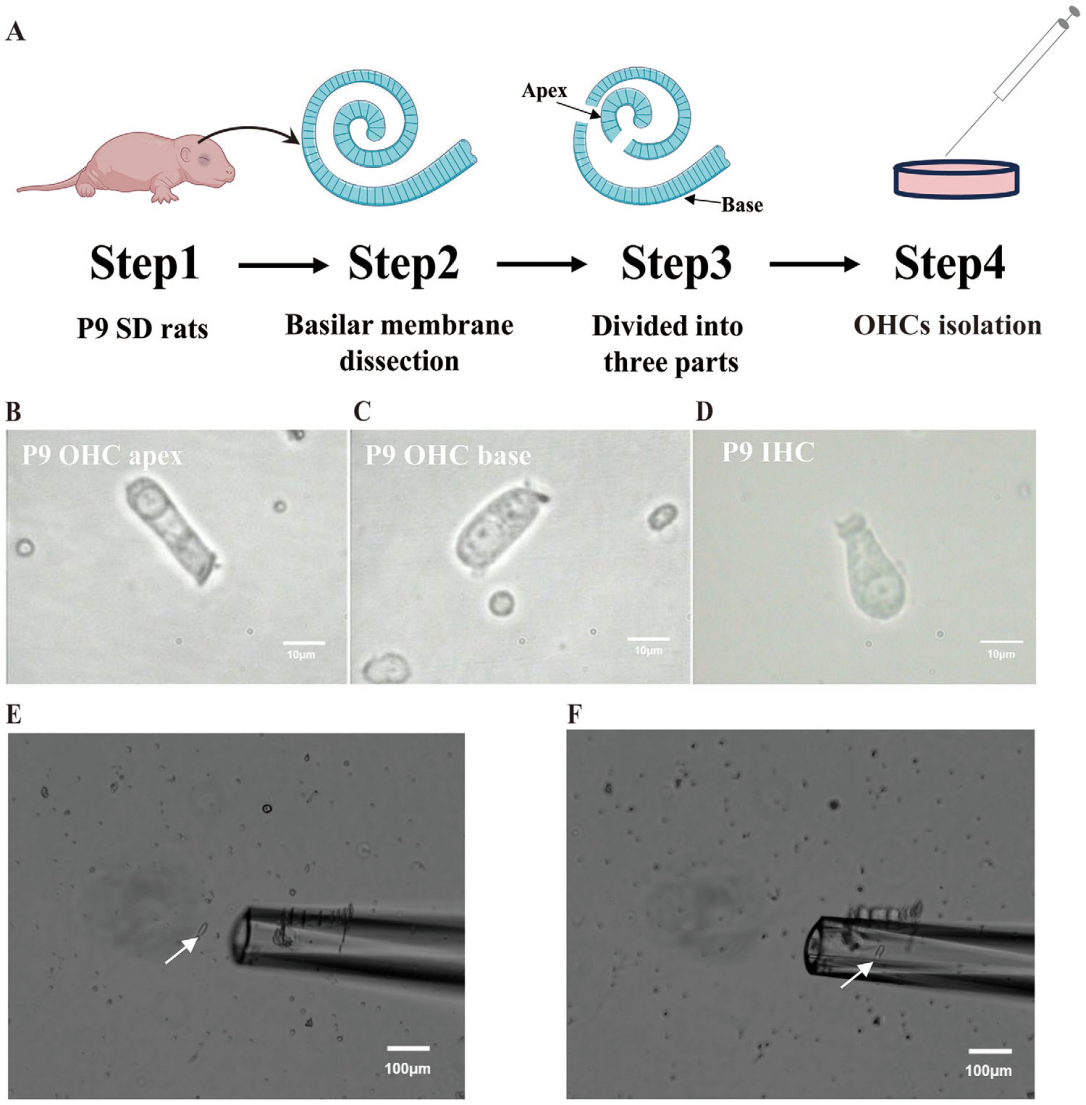
Isolation and capture of apical and basal OHCs from P9 SD rats. A) Steps for isolating apical and basal OHCs. B) Image of an isolated apical OHC with a cylindrical shape and stereocilia. C) Image of an isolated basal OHC with a cylindrical shape and stereocilia, but shorter in length compared to apical OHCs. D) IHCs are typically flask‐shaped, and the cuticular plate is set at an angle to the main axis of the cell. E) Capillary glass pipette approaching an isolated OHC (white arrow). F) An OHC was drawn into the pipette. Bar = 10 µm in B, C, and D. Bar = 100 µm in E and F.

According to previous research results, the degree of cochlear ossification is an important factor affecting the number of isolated single‐free OHCs.^[^ ^14^ ^]^ Once cochlear ossification is complete, it is difficult to isolate single‐free OHCs from the basal turn. Compared with mouse cochleae, rat cochleae yield a higher number of free OHCs. Therefore, we collected the apical and basal OHCs of P9 Sprague–Dawley rats (SD rats) for scRNA‐seq. After the cochlear basilar membrane was dissected, the hook region was removed, and the basilar membrane was divided into the apical, middle, and basal parts (**Figure** **1**A). After digestion with collagenase IV, a microsyringe was used to triturate the samples gently to isolate OHCs. The number of isolated single‐free OHCs is shown in Table S1 (Supporting Information). Micromanipulation was used to capture three groups of 30 OHCs each from the apex and base for subsequent sequencing.

### 2.2 Single‐Cell Transcriptomic Characterization of Apical and Basal OHCs

**Figure 2:**
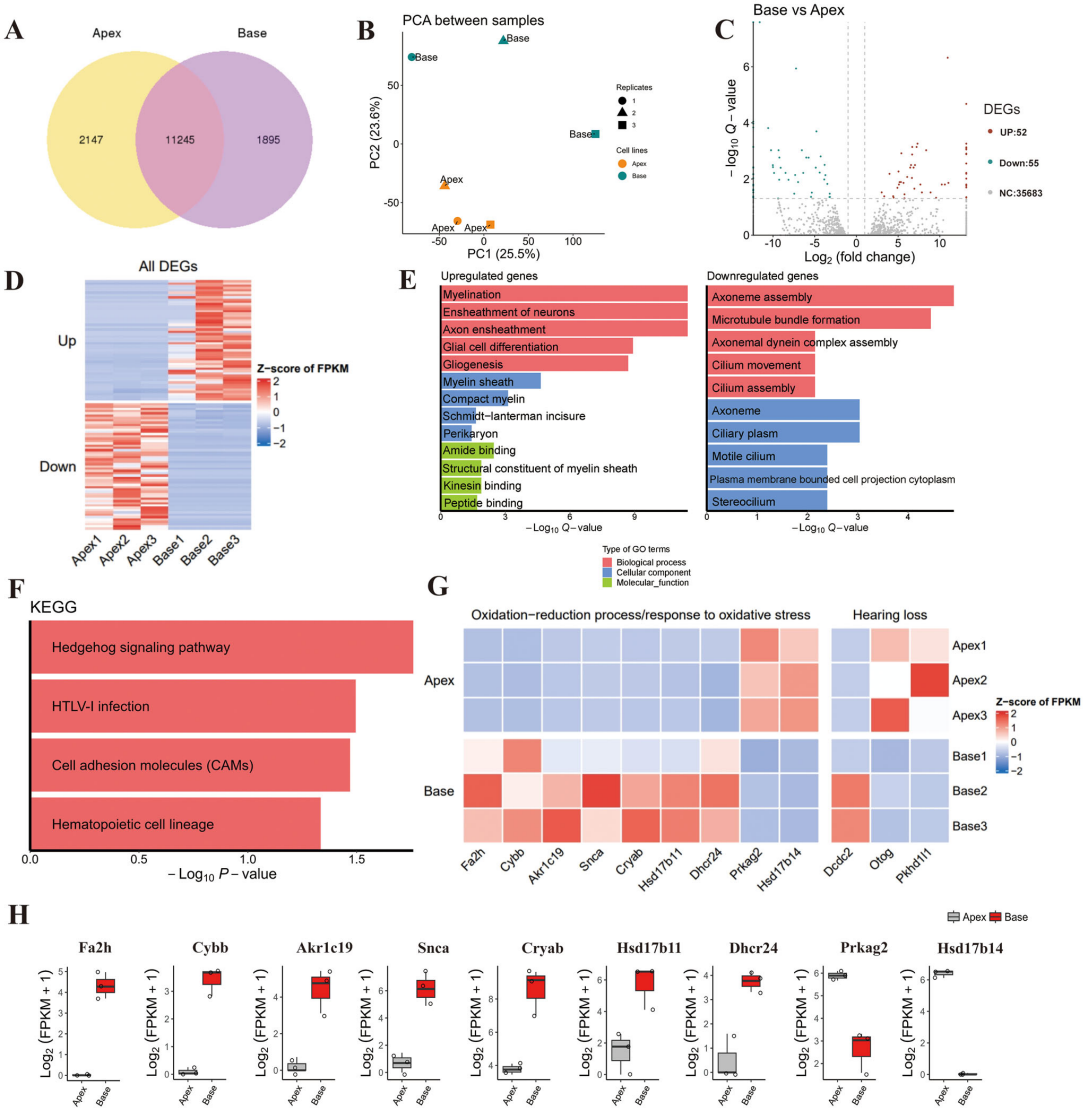
Single‐cell transcriptomic characterization of apical and basal OHCs. A) Venn diagram showing the number of genes expressed in apical and basal OHCs. B) PCA of all three replicates of apical and basal OHC populations. C) Volcano plot showing the differentially expressed genes in apical and basal OHCs. Up: genes are upregulated in the basal OHCs; Down: genes are downregulated in the basal OHCs; NC: no change, indicating the genes are not significantly different between the basal and apical OHCs. D) Heatmap showing differences in the expression of differentially expressed genes as relative expression levels. E) GO analysis of genes differentially expressed in apical and basal OHCs. F) KEGG pathway analysis of genes that were differentially expressed in apical and basal OHCs. G) Genes related to oxidation–reduction process, oxidative stress, and hearing loss in different groups. H) The expression levels of the nine genes related to oxidation–reduction and oxidative stress processes.

Single‐cell RNA sequencing revealed a total of 13392 transcripts in the apical OHCs and 13140 transcripts in the basal OHCs. Among them, 11245 transcripts were expressed in both the apical and the basal OHCs. The results also revealed that 2147 and 1895 transcripts were uniquely expressed in apical OHCs and basal OHCs, respectively (**Figure** **2**A). Principal component analysis (PCA) revealed significant differences in the transcriptome levels of the apical and basal OHCs. The reproducibility of the data for the apical OHCs was particularly good; however, the transcriptome data of the basal OHCs were highly heterogeneous, suggesting that basal OHCs are more sensitive to external stimuli (Figure 2B).

There were 107 genes differentially expressed between apical and basal OHCs, 52 of which were upregulated in basal OHCs relative to apical OHCs and 55 of which were downregulated in basal OHCs relative to apical OHCs (Figure 2C,D; Table S2, Supporting Information). Gene Ontology (GO) enrichment analysis of the differentially expressed genes revealed that the biological processes, cellular components, and molecular functions of the apical and basal OHCs involved mainly neural development, microtubule formation, and cilia assembly (Figure 2E). Kyoto Encyclopedia of Genes and Genomes (KEGG) pathway analysis revealed that Hedgehog signaling pathway activation was the top enriched term for the differentially regulated gene sets (Figure 2F).

We also identified nine differential expressed genes related to oxidative damage (Figure 2G,H). The expression of Cybb, which is known as NOX2, significantly differed between apical and basal OHCs. Previous studies have confirmed that Nox2 is an important molecule in oxidative stress mechanisms;^[^ ^16^, ^17^, ^18^ ^]^ therefore, we chose to investigate the relationship between the NOX2 gene and the vulnerability of high‐frequency OHCs.

Deafness‐related genes, which can cause syndromic and nonsyndromic deafness, can be found at http://hereditaryhearingloss.org. Among these genes, Dcdc2, Pkhd1l1, and Otog presented differences in expression between apical and basal turn OHCs (Figure 2G).

### 2.3 NOX2 Expression Differed Significantly in Apical and Basal OHCs After Noise and Neomycin Injury

**Figure 3:**
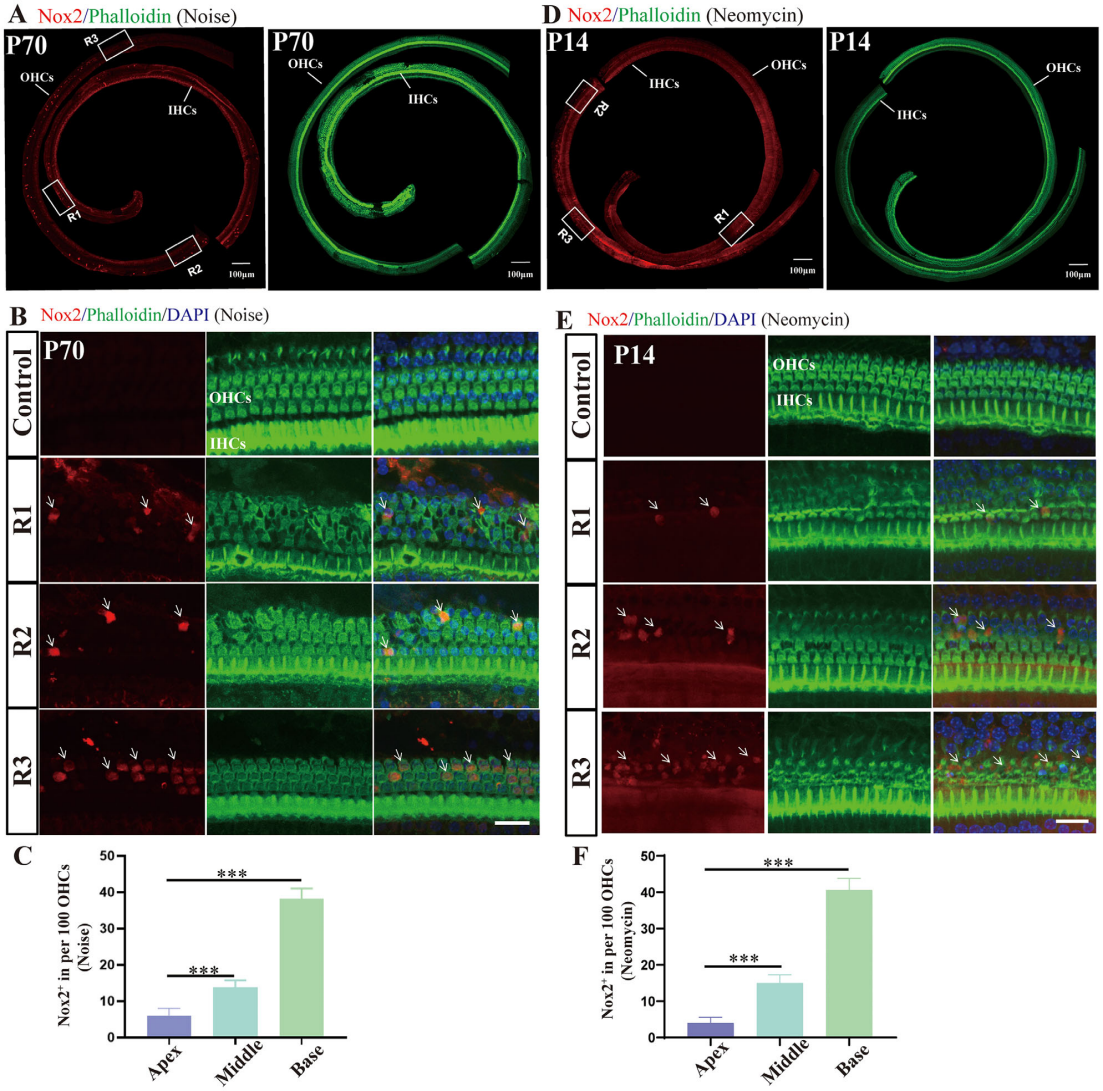
After noise and neomycin injury, the expression of NOX2 significantly differed from the apex to the base. A) 14 days after noise exposure (105 dB, 2 h), immunofluorescence staining of the basilar membrane of the mouse cochlea was performed. The number of Nox2 (red)‐positive OHCs significantly differed was significantly different from the apex to the base. B) Magnified laser confocal photo of the three areas (R1, R2, and R3) in panel A. Phalloidin (green) was used to label hair cells. White arrows indicate Nox2‐positive OHCs. C) Quantitative analysis of panels A and B. Data are presented as the mean ± standard deviation. (*n* = 5) D) After neomycin treatment, immunofluorescence staining of the basilar membrane showed that the number of Nox2‐positive OHCs increased gradually from the apex to the base. E) Magnified laser confocal photo of the three areas (R1, R2, and R3) in panel D. White arrows indicate Nox2‐positive OHCs. F) Quantitative analysis of panels D and E. Data are presented as the mean ± standard deviation. (*n* = 5). For all experiments, ^*^ *p <* 0.05, ^**^ *p<* 0.01, and ^***^ *p <* 0.001. Bar = 20 µm in B and E. Bar =100 µm in A and D.

Consistent with our previous findings in SD rats, before exposure to noise or ototoxic drugs, Nox2 was expressed at a low level in the OHCs of the cochlear basilar membrane of C57 mice, with no difference between apical and basal OHCs (Figure S1, Supporting Information).^[^ ^19^ ^]^ First, we focused on the relationship between NOX2 and the vulnerability of basal OHCs after noise exposure. 14 days after noise exposure (105 dB, 2 h), immunofluorescence results revealed that the number of Nox2‐positive OHCs gradually increased from the apical turns to basal turns (**Figure** **3**A). The number of Nox2‐positive OHCs at the base was significantly greater (8.15 times greater) than that at the apex (Figure 3B,C). Interestingly, in the hook area, which is located at the base of the basilar membrane, no Nox2‐positive OHCs were found.

We further investigated the correlation between NOX2 expression in OHCs and high‐frequency OHCs vulnerability after neomycin injury. First, we constructed a mouse neomycin injury model. After neomycin injury in C57BL/6J mice, the degree of damage to OHCs gradually increased from the apex to the base, and the number of Nox2‐positive OHCs also gradually increased from the apex to the base (Figure 3D,E). Statistical analysis revealed that the number of Nox2‐positive cells in basal OHCs was 28 times greater than that in apical OHCs (Figure 3F).

### 2.4 NOX2 Knockout Significantly Protected Mice from Hearing Loss and Hair Cell Damage Caused by Noise or Neomycin

**Figure 4:**
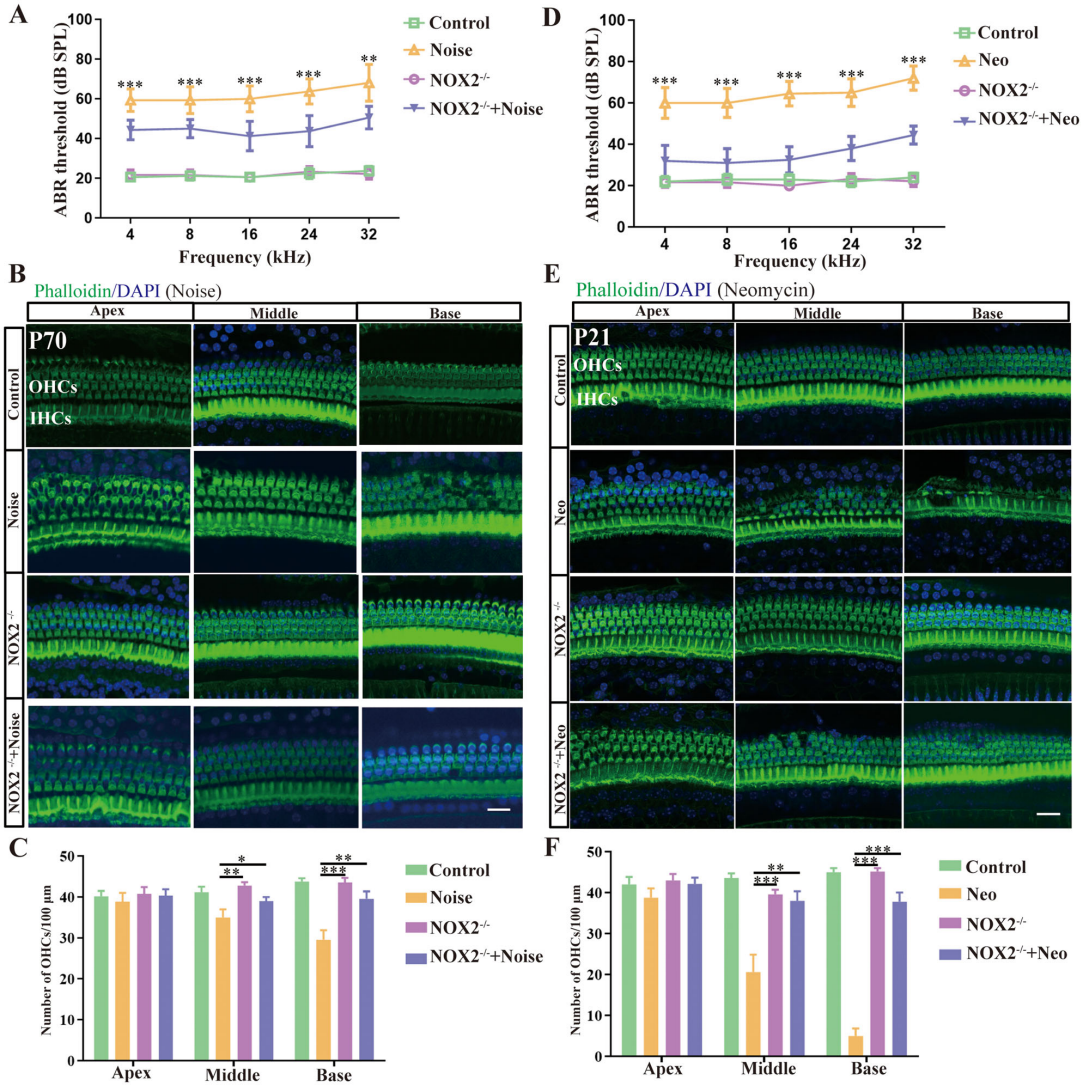
After noise and neomycin exposure, NOX2 knockout significantly reduced hearing loss and OHCs loss. A) ABR thresholds of the four groups of mice (control, noise, NOX2^−/−^and NOX2^−/−^+ noise) at frequencies of 4, 8, 16, 24, and 32 kHz. NOX2 knockout significantly reduced noise‐induced increases in hearing thresholds. B) Immunofluorescence staining of the cochlear basilar membrane in different treatment groups (phalloidin, green). Compared with that in the noise group, there was a significant reduction in the loss of OHCs in the NOX2^−/−^ + noise group. C) Quantification of panel B. Data are presented as the mean ± standard deviation (*n* = 6). D) ABR thresholds of the four groups of mice (control, neomycin, NOX2^−/−^ and NOX2^−/−^ + neomycin) at frequencies of 4, 8, 16, 24 and 32 kHz. NOX2 knockout significantly reduced the increase in hearing threshold caused by neomycin stimulation. E) Immunofluorescence staining in different treatment groups (phalloidin, green). Compared to the neomycin treatment group, there was a significant reduction in the loss of OHCs in the NOX2^−/−^ + neomycin group. F) Quantification of panel E (n = 6). Bar= 20 µm. For all experiments, ^*^ *p <* 0.05, ^**^ *p* < 0.01, and ^***^ *p* < 0.001.

To investigate the role of Nox2 in C57 mouse OHCs, we used NOX2 knockout (NOX2^−/−^) mice (Figure S2, Supporting Information). First, we investigated whether NOX2 knockout could alleviate hearing loss induced by noise exposure. No obvious abnormalities in the morphology or number of OHCs in the cochlea or hearing function were detected in NOX2 knockout mice. Wild‐type (WT) C57 mice and NOX2^−/−^ mice were exposed to noise (105 dB, 2 h). 14 days after noise exposure, the auditory brainstem response (ABR) of the mice was evaluated. The results showed that NOX2 knockout significantly alleviated noise‐induced hearing loss at 4, 8, 16, 24, and 32 kHz (**Figure** **4**A). The DPOAE results also indicated that NOX2 knockout significantly ameliorates noise‐induced OHCs damage. After noise exposure, the mean DPOAE amplitudes of WT mice were significantly lower than those of NOX2 knockout mice (Figure S3A, Supporting Information). Immunofluorescence staining of the cochlea revealed that the area of hair cell damage was concentrated at the basal turn, with sporadic loss of middle OHCs and no obvious loss of OHCs at the apex (Figure 4B). Knocking out NOX2 significantly reduced the loss of OHCs caused by noise. The statistical results revealed that knocking out NOX2 alleviated the loss of middle and basal OHCs (Figure 4C).

Similarly, we investigated whether NOX2 knockout could alleviate neomycin‐induced OHC damage. ABR testing revealed that NOX2 knockout significantly alleviated neomycin‐induced hearing loss at 4, 8, 16, 24, and 32 kHz (Figure 4D). DPOAE results also demonstrated that NOX2 knockout significantly mitigates neomycin‐induced outer hair cell damage (Figure S3B, Supporting Information). After neomycin was used to injure the cochleae, the damage to the OHCs gradually increased from the apex to the base. No obvious loss of OHCs was observed at the apex, but the loss of middle and basal OHCs was substantial. Although NOX2 knockout failed to fully prevent the OHC damage caused by neomycin, it significantly reduced the loss of middle and basal OHCs (Figure 4E). The statistical results also revealed that knocking out NOX2 reduced the loss of middle and basal OHCs and had the most significant protective effect on basal OHCs (Figure 4F).

Furthermore, we dissected the cochlear basilar membranes of WT mice and NOX2^−/−^ mice at postnatal day 3 (P3) and cocultured them with 1 mm neomycin for 24 h. Compared with the WT, NOX2 knockout conferred significant protection against neomycin‐induced OHC loss in vitro (Figure S3, Supporting Information). The above results showed that NOX2 knockout had a protective effect against hair cell loss and hearing loss caused by neomycin and noise and that the protective effect on basal OHCs was most significant.

### 2.5 NOX2 Knockout Reduced Neomycin‐Induced Mitochondrial ROS Production and Inhibited Apoptotic Pathway Activation

**Figure 5:**
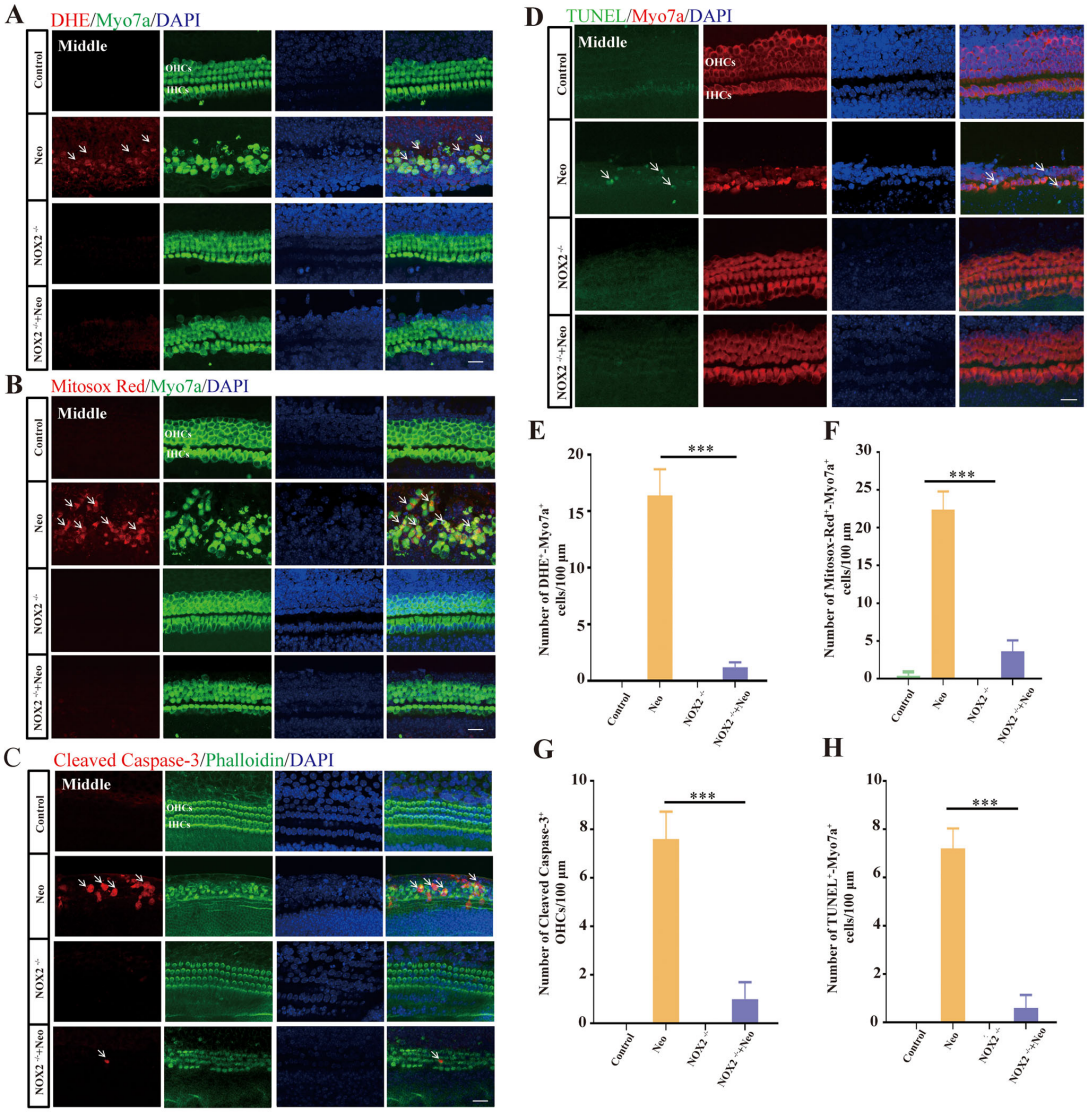
NOX2 knockout reduces neomycin‐induced mitochondrial ROS production and inhibits neomycin‐induced apoptotic pathway activation. A) Immunofluorescence staining of in vitro cochlear basilar membrane cultures from four groups of mice (middle turn; DHE, red; myosin 7a, green). Compared with WT mice whose basilar membrane was damaged by neomycin, NOX2 knockout significantly reduced the number of DHE‐positive OHCs (White arrows). B) Immunofluorescence images of MitoSOX Red levels in the cochlear basilar membrane in different treatment groups (middle turn; myosin 7a, green). NOX2 knockout significantly reduced MitoSOX Red levels in OHCs after neomycin injury. White arrows indicate Mitosox Red‐positive OHCs. C) Immunofluorescence images of cleaved caspase‐3 (red) expression levels in OHCs of mice in different treatment groups (middle turn; phalloidin, green). White arrows indicate cleaved caspase‐3‐positive OHCs. D) Immunofluorescence images of TUNEL (green) levels in OHCs of mice in different treatment groups (middle turn; myosin 7a, red). NOX2 knockout significantly reduced the neomycin‐induced increase in the number of apoptotic OHCs. White arrows indicate TUNEL‐positive OHCs. E) Quantitative analysis of panel A (*n* = 5). F) Quantitative analysis of panel B (*n* = 5). G) Quantitative analysis of panel C (*n* = 5). H) Quantitative analysis of panel D (*n* = 5). Bar = 20 µm. For all experiments, ^*^ *p* < 0.05, ^**^ *p* < 0.01, and ^***^ *p* < 0.001. Data are presented as the mean ± SD.

ROS generated by Nox2 are mainly superoxide anions, and the level of superoxide anions can be detected by using DHE.^[^ ^20^, ^21^, ^22^, ^23^ ^]^ The cochlear basilar membranes of WT mice and NOX2^−/−^ mice at P3 were dissected for cochlear explant culture. Before neomycin stimulation, DHE levels were low in the basilar membrane of WT and NOX2^−/−^ mice. After neomycin treatment, the level of DHE in the basilar membrane of WT mice significantly increased. After NOX2 was knocked out, the neomycin‐induced increase in DHE significantly decreased (**Figure** **5**A). The quantitative statistical results also revealed that after NOX2 was knocked out, the neomycin‐induced increase in DHE‐positive OHCs significantly decreased (*p <*0.001) (Figure 5E).

Previous studies have shown that the ROS produced by NADPH oxidase can lead to mitochondrial dysfunction.^[^ ^24^, ^25^, ^26^ ^]^ Mitochondria are the main site of intracellular ROS and oxidative damage,^[^ ^27^, ^28^ ^]^ and MitoSOX Red can be used to assess mitochondrial reactive oxygen levels.^[^ ^29^, ^30^ ^]^ Before neomycin treatment, the MitoSOX Red level was low in the basilar membrane of both WT mice and NOX2 knockout mice. After neomycin injury, the level of MitoSOX Red in the basilar membrane of WT mice significantly increased; in contrast, knocking out NOX2 significantly reduced the level of MitoSOX Red (Figure 5B). The quantitative statistical results revealed that knocking out NOX2 significantly reduced the neomycin‐induced increase in the number of MitoSOX Red‐positive OHCs (Figure 5F).

ROS‐induced damage to OHCs activates apoptotic pathways.^[^ ^6^, ^31^, ^32^, ^33^, ^34^, ^35^ ^]^ When not treated with neomycin, cleaved caspase‐3‐positive cells were not observed among the OHCs of the basilar membrane of WT or NOX2^−/−^ mice. After neomycin injury, the number of cleaved caspase‐3‐positive apoptotic cells in the basilar membrane of WT mice significantly increased, and knocking out NOX2 significantly reduced neomycin‐induced OHC apoptosis (Figure 5C). The TUNEL staining results were consistent with the cleaved caspase‐3 results (Figure 5D). Additionally, quantitative statistical results also revealed that the neomycin‐induced increase in the number of apoptosis‐positive OHCs was significantly reduced in NOX2 knockout mice (Figure 5G,H).

Furthermore, we studied the effect of NOX2 knockout on neomycin‐induced OHC apoptosis in vivo. The number of cleaved caspase‐3‐positive apoptotic cells in the cochleae of WT mice significantly increased after neomycin injury. Knocking out NOX2 significantly reduced the neomycin‐induced increase in the number of cleaved caspase‐3‐positive apoptotic OHCs (Figure S4, Supporting Information). The above results confirmed that knocking out NOX2 can significantly reduce the neomycin‐induced production of mitochondrial ROS and inhibit the neomycin‐induced activation of apoptosis.

### 2.6 Knocking Out NOX2 Increased the Expression and Nuclear Translocation of Nrf2 in Cochlear OHCs

**Figure 6:**
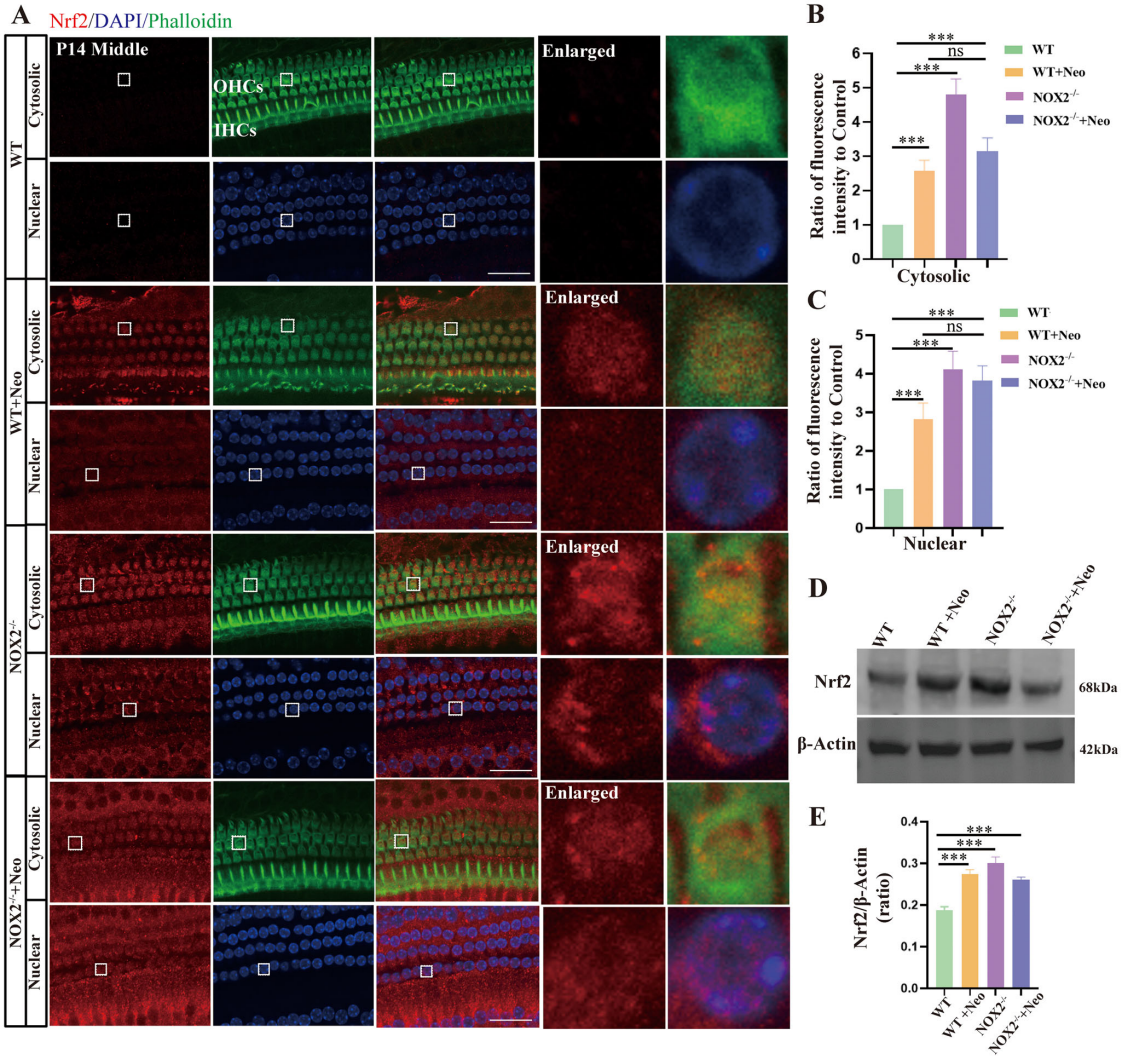
Knocking out NOX2 increased the expression and nuclear translocation of Nrf2 in cochlear OHCs. A) Immunofluorescence images of Nrf2 (red) expression levels in the cytoplasm and nucleus of OHCs of the basilar membrane in different treatment groups. The expression level of Nrf2 in the cytoplasm of OHCs significantly increased after NOX2 was knocked out, and nuclear translocation occurred (middle turns; phalloidin, green). *n* = 6. B) Semiquantitative analysis of Nrf2 in the cytoplasm of OHCs by immunofluorescence. C) Semiquantitative analysis of Nrf2 in the nucleus of OHCs by immunofluorescence. D) Representative Western blot (WB) results showed that Nrf2 expression levels in total cochlear proteins were higher in cochleae from NOX2 knockout mice than in those from WT mice. WBs were repeated three times, with three cochleae per sample. β‐Actin was used as a loading control. E) Quantitative analysis of WB results in panel D. Bar = 20 µm. For all experiments, ^*^ *p* < 0.05, ^**^ *p* < 0.01, and ^***^ *p* < 0.001. Data are presented as the mean ± SD.

Under normal conditions, oxidation and antioxidation processes in cells are in a balanced steady state; however, the balance can be disrupted by external stimulation. Nrf2 is a key transcription factor that regulates antioxidative stress and lies at the center of a complex regulatory network.^[^ ^36^, ^37^ ^]^ Therefore, we investigated the relationship between Nox2 and Nrf2 in vivo. After neomycin stimulation, Nrf2 expression in the cytoplasm of OHCs in WT mice was significantly upregulated, and Nrf2 was translocated to and accumulated in the nucleus. After NOX2 was knocked out, the expression level of Nrf2 in the cytoplasm of OHCs significantly increased, and nuclear translocation occurred. After NOX2^−/−^ mice were treated with neomycin, Nrf2 expression in the cytoplasm and nucleus of OHCs remained high (**Figure** **6**A–C). The results of the Western blot (WB) also revealed that after NOX2 was knocked out, the Nrf2 protein level was significantly higher than that in normal WT mice. These results indicate that knocking out NOX2 increased the level of Nrf2 in OHCs, thereby increasing the resistance of OHCs to external damage.

### 2.7 ML385 Abolished the Protective Effect of NOX2 Knockout on Neomycin‐Induced OHC Injury

**Figure 7:**
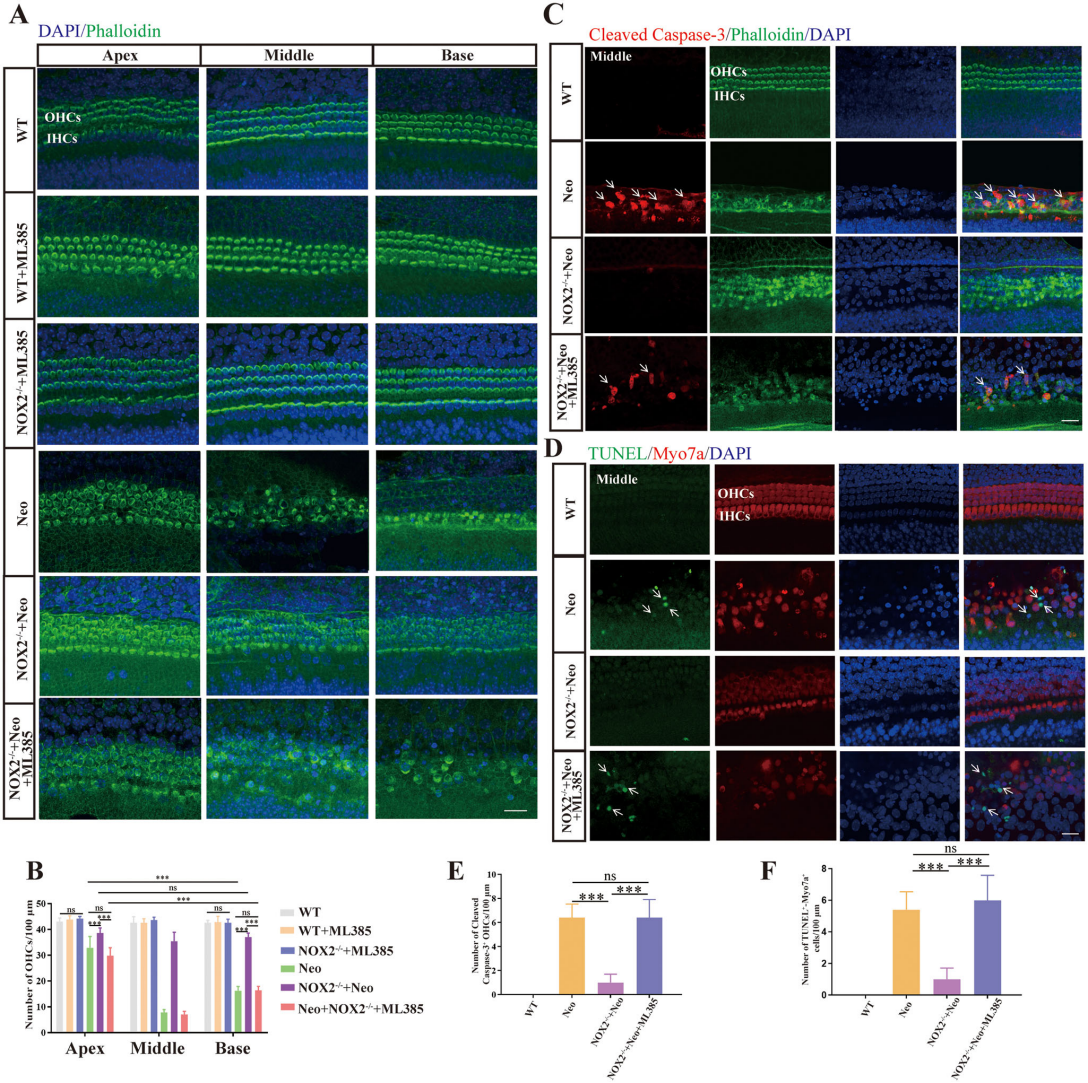
Inhibition of Nrf2 abolished the protective effect of NOX2 knockout against neomycin‐induced OHC damage. A) Immunofluorescence images of the cochlear in different treatment groups (WT, WT+ML385, NOX2^−/−^+ML385, WT +neomycin, NOX2^−/−^+neomycin, and NOX2^−/−^+neomycin +ML385). Phalloidin (green) (*n* =6). Knocking out NOX2 resulted in significant OHC protection. When OHCs of the basilar membrane of NOX2^−/−^ mice were cotreated with ML385 and neomycin, the protective effect of NOX2 knockout was completely offset. B) Statistical diagram of OHCs in different treatment groups. C) Immunofluorescence images of cleaved caspase‐3 (red) expression levels in OHCs of mice in different treatment groups (middle turn; phalloidin, green). White arrows indicate cleaved Caspase‐3‐positive OHCs. D) Immunofluorescence images of TUNEL (green) staining in OHCs of mice in different treatment groups (middle turn; myosin 7a, red). White arrows indicate TUNEL‐positive OHCs. E) Quantitative analysis of panel C (*n* = 6). F) Quantitative analysis of panel D (*n* = 6). Bar = 20 µm. For all experiments, ^*^ *p* < 0.05, ^**^ *p* < 0.01, and ^***^ *p* < 0.001. Data are presented as the mean ± SD.

Next, we verified whether the protective effect of Nox2 against neomycin‐induced OHC damage is achieved through Nrf2. ML385 is a specific Nrf2 inhibitor.^[^ ^38^, ^39^ ^]^ The concentration of ML385 used in this study was 5 µm, as supported in the literature.^[^ ^40^ ^]^ The cochlear basilar membranes of WT mice and NOX2^−/−^ mice at P3 were dissected for cochlear explant culture. ML385 treatment alone had no significant toxic effects on the OHCs of the basilar membrane in WT or NOX2^−/−^ mice. After treatment with neomycin alone, the loss of OHCs in WT mice gradually increased from the apex to the base, whereas knocking out NOX2 had a significant protective effect on OHCs and reduced OHC death (Figure 7A). However, the protective effect of NOX2 knockout was completely abolished when OHCs of the basilar membrane of NOX2^−/−^ mice were cotreated with ML385 and neomycin; the counting results confirmed this finding (**Figure** **7**B).

Moreover, we used cleaved caspase‐3 and TUNEL staining to detect apoptotic OHCs in each group. After neomycin injury, the number of cleaved caspase‐3‐ and TUNEL‐positive apoptotic cells significantly increased, whereas NOX2 knockout significantly reduced OHC apoptosis. When OHCs of the cochlear basilar membrane from NOX2^−/−^ mice were cotreated with ML385 and neomycin, the number of cleaved caspase‐3‐ and TUNEL‐positive apoptotic cells increased significantly (Figure 7C‐F). These results showed that ML385 completely reversed the inhibitory effect of NOX2 knockout on the increase in cleaved caspase‐3‐ and TUNEL‐positive OHCs and that the protective effect of NOX2 knockout against neomycin‐induced OHC damage was dependent on Nrf2.

### 2.8 Nox2 is a Target of Ginsenoside Rg1, Which has a Protective Effect on Neomycin‐Induced Hearing Loss

**Figure 8:**
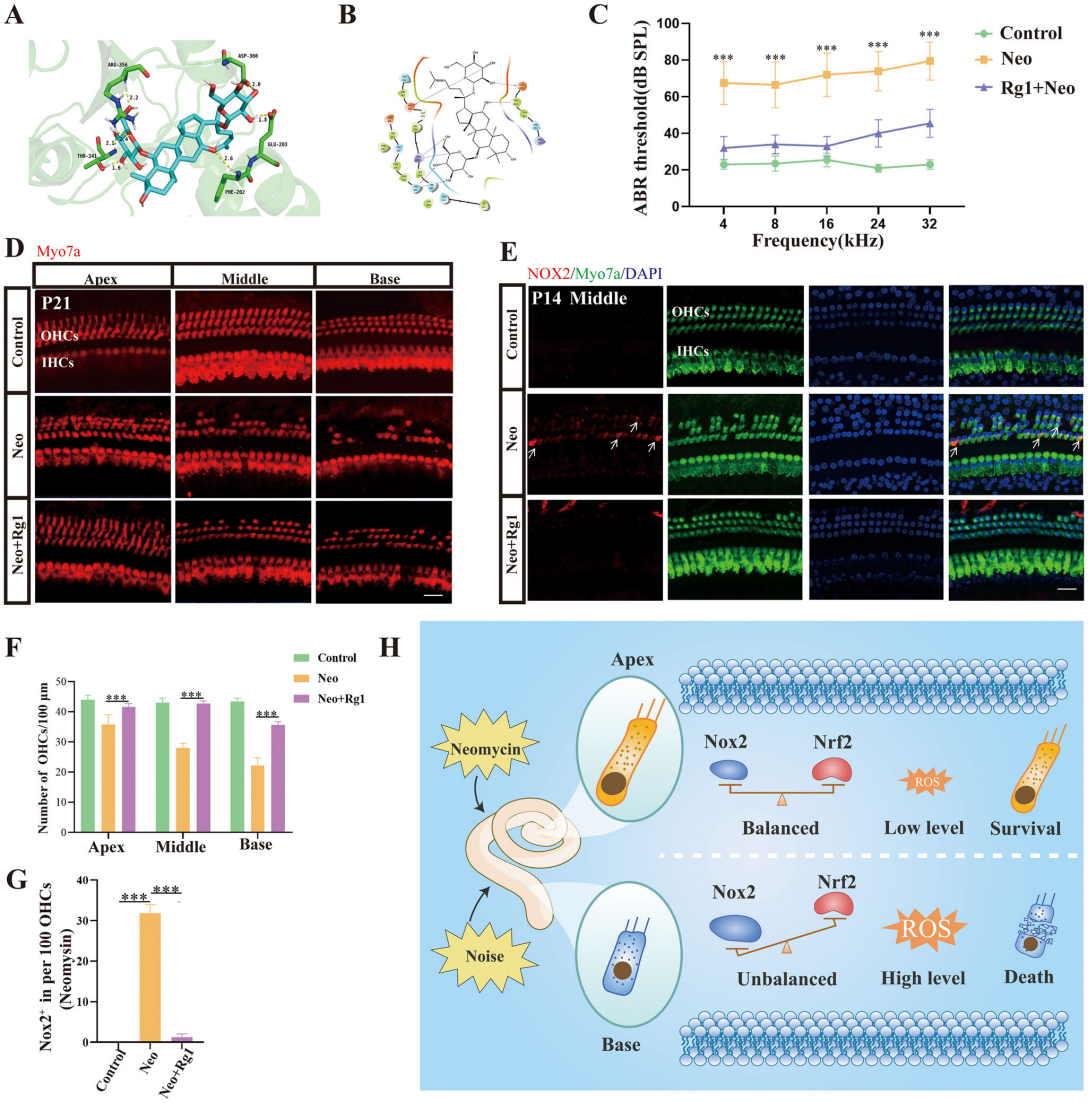
Ginsenoside Rg1 has a significant protective effect against neomycin‐induced hearing loss. A,B) Virtual drug screening revealed that ginsenoside Rg1 can form 7 hydrogen bonds with the human NOX2 protein. C) ABR thresholds measured in three groups of mice (control, neomycin, and Rg1+neomycin) tested at 4, 8, 16, 24 and 32 kHz. Ginsenoside Rg1 significantly reduced the neomycin‐induced increase in hearing thresholds in mice. D) Immunofluorescence staining of the cochlear basilar membrane in different treatment groups (myosin 7a, red). E) Immunofluorescence staining of NOX2 (red) in the cochlear basilar membrane in different treatment groups (myosin 7a, green). Ginsenoside Rg1 significantly reduced OHC loss. Bar = 20 µm. F) Count results for panel D (*n* = 6). G) Count results for panel E (*n* = 6). For all experiments, ^*^ *p* < 0.05, ^**^ *p* < 0.01, and ^***^ *p* < 0.001. Data are presented as the mean ± SD. H) Schematic diagram of NOX2 participation in oxidation equilibrium in apical and basal OHCs.

We attempted to verify the clinical value of Nox2 as a target for the treatment of neomycin‐induced SNHL. Virtual drug screening has become a new technology in recent years and involves the use of computers to analyze the molecular functional characteristics of a given protein receptor, screen drugs or other small molecule compounds in the database, and identify candidate bioactive molecules.^[^ ^41^, ^42^ ^]^ We used this technology to screen molecules in a natural compound library that targets human Nox2. Through a comprehensive evaluation of the binding force and safety of small‐molecule compounds, we found that ginsenosides Rg1 and Nox2 bind stably (**Figure** **8**A,B) Previous reports in the literature have shown that the ginsenoside Rg1 plays an important role in antioxidation and neuroprotection; however, its role in SNHL has not been studied.^[^ ^43^ ^]^

First, we investigated whether ginsenoside Rg1 has a protective effect against neomycin‐induced hearing loss in mice. ABR testing revealed that ginsenoside Rg1 significantly alleviated neomycin‐induced hearing loss at 4, 8, 16, 24, and 32 kHz (Figure 8C). Compared with neomycin, ginsenoside Rg1 significantly reduced the neomycin‐induced loss of OHCs; in particular, it had a significant protective effect on basal OHCs (Figure 8D,F). In addition, ginsenoside Rg1 significantly reduced the neomycin‐induced increase in the number of Nox2‐positive OHCs (Figure 8E,G). These findings indicate that ginsenoside Rg1 exerts a protective effect on OHCs by inhibiting Nox2. The above results demonstrate that ginsenoside Rg1 has a protective effect on neomycin‐induced OHC damage, thereby preliminarily verifying that Nox2 is an intervention target with potential clinical application value.

## 3 Discussion

High‐frequency OHCs vulnerability in the cochlea is a common phenomenon where basal OHCs are more susceptible to damage than apical OHCs. Across different species, pathogenic factors, and both in vitro and in vivo experiments, the loss of hair cells is greater at the base and less at the apex.^[^ ^44^ ^]^ In this study, we identified NOX2, a differentially expressed gene associated with oxidative damage, through single‐cell RNA sequencing of apical and basal OHCs. In neomycin‐induced and noise‐induced injury models, Nox2‐positive OHCs gradually increased from the apex to the base, which is consistent with the finding that basal OHCs are more susceptible to damage.

NOX2 is a member of the NOX oxidase family and was first discovered in phagocytes, in which it performs host defense functions. Nox2 transfers electrons across the plasma membrane to generate ROS, which plays a variety of physiological and pathological roles. NOX3 is highly expressed in the inner ear. The production of ROS by NOX3 may be related to deafness, and knocking out NOX3 can alleviate noise‐induced hearing loss.^[^ ^45^, ^46^ ^]^ Cisplatin can induce the expression of NOX1 and NOX4 in the HEI‐OC1 cell line, and NADPH oxidase inhibitors can reduce cisplatin‐induced ROS generation and apoptotic cell death.^[^ ^47^ ^]^ However, other NOX family members in the cochlea have not been well studied.

Our results suggest that Nox2‐derived ROS are common factors contributing to OHC death and dysfunction following neomycin or noise exposure. Although there are some similarities and differences in the pathogenesis of noise‐induced hearing loss and drug‐induced hearing loss, excessive production of ROS in the cochlea plays a core role in the etiology.^[^ ^48^ ^]^ The severity of noise‐induced hearing loss (NIHL) is related to the intensity, frequency, and duration of noise stimulation. Excessive acoustic stimulation can cause cochlear mitochondrial overdrive, reduce cochlear blood flow, and dysregulate calcium homeostasis, thereby promoting excessive production of ROS and mitochondrial damage.^[^ ^49^ ^]^ Moreover, noise can also trigger an acute inflammatory response, leading to further cochlear damage. After aminoglycosides enter hair cells, they can be transported to lysosomes through a vesicle‐mediated process, where they accumulate and cause lysosomal rupture. Aminoglycosides can also accumulate in mitochondria, causing impaired mitochondrial function.^[^ ^50^ ^]^

Aminoglycoside antibiotics primarily damage OHCs but can also cause ribbon synapses in inner hair cells. NIHL is related not only to damage to outer hair cells but also to damage to synapses and spiral neurons. Excessive production of ROS can cause irreversible damage to DNA, proteins, lipids, and other macromolecules. Ultimately, a series of stimuli, such as inflammation and ROS, can trigger cell necrosis or programmed cell death. After cochlear damage from ototoxic drugs or noise, presynaptic ribbons degrade before OHCs die.^[^ ^50^ ^]^ Some of the remaining OHCs also become dysfunctional.^[^ ^51^, ^52^, ^53^ ^]^ This phenomenon also explains why the ABR threshold increases and why auditory function is impaired in the apical turn, where the survival rate of outer hair cells is higher. Mice can hear sounds over a wide frequency range, up to 64–68 kHz, which exceeds the range that our audiometric equipment can detect.^[^ ^54^ ^]^ This has limited our understanding of high‐frequency hearing loss to some extent.

To our knowledge, NOX2 is the first gene identified to be differentially involved in apical and basal OHC vulnerability. In this study, we thoroughly investigated the relationship between NOX2 and the high‐frequency OHCs vulnerability of the cochlea. An important reason for the moderate correlation between noise‐induced hearing loss and hair cell loss is that noise not only causes OHC death but also impairs the function of some surviving OHCs.^[^ ^55^ ^]^ We found that 14 days after noise exposure, many OHCs still strongly expressed NOX2, with NOX2 expression mainly concentrated in the basal OHCs. Although these OHCs survive, their function may be impaired, which manifests as more severe hearing loss. Interestingly, in the basilar membrane hook region, the immunofluorescence level of Nox2 in OHCs was significantly reduced, which is consistent with the findings of some studies showing that hair cells in the hook region are resistant to damage.^[^ ^44^ ^]^ There was a clear correlation between neomycin‐induced hearing loss and OHC loss. The loss of OHCs increased gradually from the apex to the base, and the NOX2 expression pattern was completely consistent with this damage pattern. These results show that NOX2 is clearly associated with the vulnerability of basal OHCs.

Noise, aging, drugs, and other factors can disrupt the balance of oxidation and antioxidation processes in OHCs. We revealed that NOX2 knockout has a significant protective effect against hearing loss and hair cell damage caused by noise or neomycin, and this protective effect is mediated through Nrf2. Nrf2 is a key regulator of antioxidant responses. Elevated NOX4 levels can inhibit Nrf2 nuclear translocation in human astrocytes.^[^ ^56^ ^]^ In liver cancer cells, NOX4 expression is negatively correlated with Nrf2 activity, and NOX4 deprivation activates the Nrf2 pathway.^[^ ^57^ ^]^ In another study, knocking out Nrf2 in glial neurons led to increased ROS production and increased NOX2 expression.^[^ ^58^ ^]^ In this study, we found that knocking out NOX2 increased the expression and nuclear translocation of Nrf2 in cochlear OHCs and reduced the activation of apoptotic pathways. Furthermore, the use of Nrf2 inhibitor counteracted the protective effect of knocking out NOX2 against neomycin‐induced damage, indicating that the protective effect of knocking out NOX2 against neomycin‐induced OHC damage is exerted through Nrf2.

We also found that knocking out NOX2 in vitro significantly inhibited the production of mitochondria‐derived ROS, indicating a correlation between NOX2 and mitochondrial function. Previous studies reported that NOX2 overexpression increased mitochondrial ROS production and led to mitochondrial dysfunction in placental blood vessels, which is consistent with our findings.^[^ ^59^ ^]^ In another study, in placental preeclampsia cells, upregulated NOX2 induced trophoblast ferroptosis through the STAT3/GPX4 pathway, leading to increased levels of ROS and lipid peroxidation and impaired mitochondrial function, whereas the inhibition of NOX2 alleviated placental cell ferroptosis and mitochondrial dysfunction.^[^ ^60^ ^]^ However, it's not just that NOX2 expression can regulate mitochondrial function, mitochondria can in turn affect the function of NADPH oxidase. There is evidence that mitochondrial ROS can promote the activation of NADPH oxidase in phagocytes and that Nox2 may be a specific target of mitochondrial ROS.^[^ ^61^ ^]^ Nrf2 is also involved in promoting mitochondrial quality control and regulating basic mitochondrial functions, including energy production, ROS generation, calcium signaling, and the induction of cell death signals.^[^ ^62^ ^]^ Therefore, in OHCs damaged by oxidative stress, the balance between Nox2 and Nrf2 is disrupted, leading to the production and dysfunction of mitochondrial ROS and ultimately causing OHC deaths (Figure 8H).

Our study revealed that NOX2 is significantly associated with differences in the vulnerability of apical and basal OHCs under two different injury conditions: noise and neomycin. However, further experiments are still needed to verify whether NOX2 affects deafness caused by other factors such as aging and cisplatin. We used a virtual drug screening method to screen therapeutic drugs targeting Nox2, aiming to verify the clinical value of NOX2 in preventing sensorineural hearing loss. Virtual drug screening revealed that ginsenoside Rg1 may target Nox2, and in vivo experiments in mice confirmed the protective effect of ginsenoside Rg1 against neomycin‐induced deafness. These experimental results confirm that Nox2 is an ideal target for the treatment of SNHL and has potential clinical application value.

Additionally, we used scRNA‐seq technology to analyze the differences in the vulnerability of apical and basal OHCs. The transcriptome represents all the RNA molecules of a cell at a specific period, including mRNAs, rRNAs, tRNAs, and other noncoding RNAs, which contain information about cell proliferation, differentiation, and metabolism.^[^ ^63^ ^]^ Guoqiang Sun established a dynamic transcriptomic landscape of mouse cochlear aging through scRNA‐seq technology and described the aging‐associated transcriptomic changes in 27 different cochlear cell types across five different time points.^[^ ^64^ ^]^ Conventional bulk RNA sequencing is difficult because the auditory organ of the inner ear is highly heterogeneous and has a very limited number of cells.^[^ ^65^ ^]^ There are several reasons for the use of P9 SD rats for scRNA‐seq. Our previous studies revealed that more isolated OHCs could be isolated from SD rats than from C57BL/6J mice; sufficient yields of basal OHCs can be isolated from P9 SD rats for downstream experiments.^[^ ^14^ ^]^ The repeatability of the data for apical OHCs was particularly good, but the reproducibility for basal OHCs was more heterogeneous. Although we strictly controlled the entire sampling process, the cochlear basilar membrane underwent collagenase digestion and subsequent mechanical separation, which served as external stimulation factors. These reproducibility differences indicate that basal OHCs are more sensitive to external stimuli, which is consistent with our experimental results. We selected SD rats and C57BL/6J mice as the research model animals. Although both are commonly used models in the field of hearing research, their different genetic backgrounds may somewhat undermine the persuasiveness of the research conclusions.

KEGG pathway analysis revealed that the activation of the Hedgehog signaling pathway, which is involved in the ability of the cochlea to distinguish corresponding sound frequencies,^[^ ^66^ ^]^ differs between the apex and base. Moreover, Dhh and Wnt7a are components of the Hedgehog signaling pathway and might also be involved in the recognition of sound frequencies. Among the differentially expressed genes in the apical and basal turns, genes related to myelination, mainly MPZ and Pmp22, appeared in the OHCs at the base. We analyzed two publicly available OHC transcriptome datasets (GSE157398; GSE56866) and identified the expression of Mpz and Pmp22 in both, further supporting their presence in OHC transcriptomes. Researchers reported that 30% of the OHCs in the apical turn were missing from the cochleae of a novel mutation in the MPZ gene (Tyr145→Ser).^[^ ^67^ ^]^ Similarly, in another patient with a missense variant (p. Thr65Ala) in the MPZ gene, there was minor scattered loss of both inner and outer auditory hair cells with mild atrophy of the stria vascularis.^[^ ^68^ ^]^ These reports suggest that mutations in the MPZ gene affect hair cells.

In summary, we conducted a detailed analysis of the differences in apical and basal OHCs through scRNA‐seq and identified NOX2 as the first gene clearly associated with the vulnerability of apical and basal OHCs. This information is highly important for a deeper understanding of the mechanism of SNHL and the vulnerability of hair cells in the high‐frequency region of the cochlea.

## 4 Experimental Section

### Animals

P9 SD rats used for scRNA‐seq of apical and basal OHCs and C57BL/6J mice used in the study were purchased from the animal experiment center of Air Force Medical University. NOX2^−/−^ mice (Cat.NO. S‐KO‐01699) were generated by Cyagen Biotechnology Company (Suzhou) on the C57BL/6J background; CRISPR/Cas9 technology was used to knock out exon 4–6 of NOX2. The identification of NOX2^−/−^ mice is shown in Figure S2 (Supporting Information). All experiments used male rats and mice. All experimental operations were conducted in accordance with the Air Force Medical University Laboratory Animal Application and Protection Management Guide and were performed according to the protocol approved by the Air Force Medical University Laboratory Animal Committee (IACUC‐20230068) and in accordance with the Guide for Care and Use of Laboratory Animals of the National Institutes of Health. Every effort was made to minimize the number of animals used and reduce animal suffering. According to the AVMA Guidelines for the Euthanasia of Animals (2020 Edition), postnatal and adult mice were euthanized following anesthesia with pentobarbital sodium. For mice undergoing ABR and DPOAE testing, cochleae were harvested through cervical dislocation under anesthesia after completion of the assessments.

### Basilar Membrane Dissection and OHC Isolation

Cochlear samples were dissected and transferred to precooled Hank's balance salt solution (HBSS, Solarbio; H1025). The basilar membrane was divided into three parts: the apical turn, middle turn, and basal turn. The apical turn and basal turn were then separately transferred to two 35‐mm Petri dishes with 1 mg mL^−1^ collagenase IV (Sigma; C4‐BIOC) diluted in Leibovitz L‐15 culture medium (Sigma, L4386) at room temperature for enzymatic digestion. After 10 min, the digestion medium was removed, and 1 mL of L‐15 culture media was added to each Petri dish. The L‐15 culture medium was optimized to have a pH of 7.4 and 300 mOsm to provide electrolyte balance and better conditions for the cells.^[^ ^69^ ^]^ Two tissue samples were gently triturated using a 50 µl microsyringe. After trituration was repeated, the cells were isolated, and each Petri dish was allowed to sit at room temperature for 10 min to allow the cells to submerge to the bottom of the dish. The Petri dishes were then mounted onto the stage of an inverted phase‐contrast microscope (Nikon, Model TE2000‐U) for OHC identification and collection.

### OHC Collection

For subsequent single‐cell sequencing, only normal OHCs were collected. Normal and damaged OHCs can be distinguished based on their morphological characteristics under microscopic observation. Normal OHCs exhibit a standard cylindrical morphology with nuclei positioned in the basal region of the cells. Kinocilium of apex turn OHCs were long and obvious, but kinocilium of basal turn OHCs were not so obvious. Damaged hair cells demonstrate pathological manifestations including edema, nuclear displacement, cell membrane shrinkage, particles exhibiting Brownian motion in the cytoplasm, and disappearance of birefringence.

To collect isolated basal and apical OHCs, capillary glass pipettes were fabricated from 0.64‐mm thin‐walled borosilicate tubing (Sutter Instrument Co., BF150‐86‐10) and pulled by a micropipette puller (Sutter Instrument Co., Model P‐97) to obtain diameters of 30 and 40 µm to. The suction port of the electrode holder was connected to rubber tubing, which was connected to a three‐port valve, one of which was connected to a microsyringe that provided negative pressure to suck in cells or positive pressure to blow out cells. After 5 cells were collected, the contents of the pipette were gently transferred into a 200‐µl microcentrifuge tube containing 6 µl of precooled SMART‐Seq v4 Kit cell lysis buffer supplemented with RNase inhibitor. Then the tube was immediately placed on ice to avoid RNA degeneration during further collection procedures. A total of 30 cells were collected into each sample tube from both the apical turn and the basal turn, and three biological replicates were performed for each population to minimize the effect of individual differences.

### RNA Extraction, Purification and Quantification

Total RNA was extracted and purified using TRIzol reagent (Invitrogen; 1559626). RNA degeneration and contamination were monitored using 1% agarose gel electrophoresis. RNA purity was checked using a NanoPhotometer spectrophotometer (IMPLEN), and RNA concentrations were measured using a Qubit RNA Assay Kit and a Qubit 2.0 fluorometer (Life Technologies). RNA integrity was assessed using an RNA Nano 6000 Assay Kit and an Agilent Bioanalyzer 2100 system (Agilent Technologies).

### Transcriptome Sequencing

A total of 3 µg of RNA per sample was used for cDNA synthesis. First‐strand cDNA was synthesized using a SMART‐Seq v4 Ultra Low Input RNA Kit for Sequencing (Clontech). Double‐strand cDNA libraries were generated using the NEBNext Ultra RNA Library Prep Kit for Illumina (NEB). After end‐repair was performed, NEBNext Adaptors were ligated to the cDNAs in preparation for hybridization. cDNA fragments ranging from 150–200 bp in size were selected and purified with an AMPure XP system (Beckman). After PCR amplification was performed, the quality of the resulting products was assessed using a Bioanalyzer 2100 system (Agilent Technologies). Transcriptome sequencing was carried out on an Illumina HiSeq 2000 platform (Illumina).

### Bioinformatics Analysis

Clean data (clean reads) were obtained by removing reads containing adaptors, reads containing poly‐N sequences and low‐quality reads from the raw data. The Q20, Q30, and GC contents of the clean reads were subsequently calculated. All further analyses were based on clean, high‐quality data. Bowtie v2.2.3 was used to construct the index of the reference genome, and TopHat v2.0.12 was used to align paired‐end clean reads to the *Rattus norvegicus* reference genome (http://ftp.ensembl.org/pub/release‐89/fasta/rattus_norvegicus/dna/). HTSeq v0.6.1 was used to count the number of reads mapped to each gene. FPKM values (the expected number of fragments per kilobase of transcript sequence per million base pairs sequenced) were calculated for each gene based on the length of the gene and the number of reads that were mapped to it. DESeq R package v1.18.0 was used to analyze the differential expression of genes between the two populations (apical OHCs and basal OHCs, with three biological replicates each), and statistical routines were used to identify differential expression according to the digital gene expression data using a model based on a negative binomial distribution. The Benjamini and Hochberg approach was used to adjust the resulting *p*‐values to control for the false discovery rate, adjust *p*‐value< 0.05, and absolute log2 fold‐change >1 was considered statistically significant. A gene ontology (GO) enrichment analysis of differential expression was performed using the GOseq R package and GO terms with *p <*0.05 were considered significantly enriched. KOBAS software was used to identify significant enrichment of the differentially expressed genes and to focus on genes of interest in different signaling pathways in the KEGG database. (The raw RNA sequencing data with processed data files can be downloaded from the National Center for Biotechnology Information – Gene Expression Omnibus (GEO); GEO Accession Number: GSE111302.)

### Noise Exposure

Eight‐week‐old C57 mice were individually placed in a nine‐cm diameter cage and exposed to broadband noise at 105 dB SPL (2–20 kHz) for 2 h. The mice were placed in a sound exposure chamber equipped with a speaker (Style 2450H, JBL, Los Angeles, CA, USA) driven by a power amplifier (AX‐500U, Yamaha, Maebashi, Japan). A sound level meter (TES 1350A, Taiwan, China) was used to adjust the noise levels at various locations in the noise exposure room to ensure the consistency of noise exposure.

### Drug Administration

C57 mice in the neomycin‐treated group were subcutaneously injected with neomycin at a dose of 200 mg kg^−1^ daily from P7 to P13 C57 mice in the ginsenoside Rg1 group were injected subcutaneously with the same dose of neomycin (SIGMA, N6386‐256) every day from P7 to P13 and then were immediately injected intraperitoneally with ginsenoside Rg1(Solarbio, IG0240) at a dose of 20 mg kg^−1^. Ginsenoside Rg1 was dissolved in DMSO to prepare a storage solution according to the instructions and then prepared as a working solution with physiological saline. The normal control group was injected subcutaneously with the same volume of sterile saline or vehicle.

### Auditory Brainstem Response (ABR)

The mice were intraperitoneally injected with sodium pentobarbital (50 mg kg^−1^) and placed on a constant‐temperature heating pad in a soundproofed and electrically shielded anechoic room. Frequency‐specific auditory responses were measured using the Tucker–Davis Technology RZ6 system (Tucker–Davies Technologies, Gainesville, FL, USA). The electrodes were placed in the middle suture of the mouse's skull, the mastoid area behind the ear, and the subcutaneous part of the mouse's tail (ground). TDT System III hardware and SigGen/Biosig software were used to present the stimuli (15 ms duration tone bursts with a 1 ms rise–fall time), and the response was recorded.

The ABR filter settings: high‐pass filter = 300 Hz, low‐pass filter = 3 kHz. ABR was measured at 4, 8, 16, 24, and 32 kHz. The threshold for each frequency was determined by decreasing the intensity in 10 dB intervals and then decreasing the threshold in 5 dB steps until no systematic response was detected. ABR wave I was monitored to assess the threshold. The threshold was estimated between the lowest stimulus level at which a response was observed and the highest stimulus level at which no response was observed. All ABR measurements were performed by the same experienced experimenter. The thresholds were assigned by an expert who was blinded to the various experimental conditions.

### Distortion Product Otoacoustic Emissions (DPOAE)

The TDT workstation was utilized to capture DPOAE recordings. A pair of speakers, linked to the ear canal on the same side, produced two primary tones simultaneously, maintaining an f2/f1 ratio of 1.2, and these tones were delivered into the ear canal via specially designed rigid plastic conical tips. Audio frequencies ranging from 4 to 32 kHz, each lasting 84 milliseconds, were presented. The sound pressure levels for f1 and f2 were set to 75 and 65 dB SPL, respectively. The ER‐10B microphone system (Etymotic Research, Inc., Elk Grove Village, IL, USA) managed the collection of responses. The RZ6 D/A converter processed the analog‐to‐digital conversion. The signals underwent analysis through the application of fast Fourier transformation.

### Cochlear Basilar Membrane Culture and Drug Treatment

The mouse cochlear basilar membrane was isolated from P3 mice and cultured according to previous methods.^[^ ^70^ ^]^ After being cultured in an incubator (37 °C, 5% CO2) for 24 h, neomycin (1 mm) was added to damage the hair cells for 24 h. ML385(GLPBIO; GC19254) was used at a final concentration of 5 µm for 24 h to investigate the effects of Nrf2 inhibition on cochlear explant culture. ML385 was dissolved in DMSO to prepare a storage solution according to the instructions and then prepared as a working solution with physiological saline.

### Immunofluorescence Staining and Semiquantitative Analysis

The cochlear of the mice treated as described above were removed and fixed with 4% paraformaldehyde at 4 °C for 8 h. After decalcification by soaking in 10% EDTA, the basilar membrane was removed. After being washed with 1× PBS (Cytiva; SH30256.01), the cells were treated with 1% Triton X‐100 (Biosharp, BS084) at room temperature for 10 min and blocked with 5% BSA at 37 °C for 45 min. Primary antibodies were diluted with an antibody diluent. Fifty microliters of diluted antibodies were added to each cochlear tissue sample. After incubation at 4 °C for 48 h, the samples were washed with 1 × PBS, a fluorescent secondary antibody was added, the samples were wrapped with tin foil in the dark, incubated at room temperature for 24 h, rinsed with 1× PBS, and incubated with DAPI (1:1000; Sigma; 28718‐90‐3) or phalloidin (1:200; Abcam; ab176753). After the samples were washed, they were placed on a slide and observed under a laser confocal microscope. ImageJ (Fiji; 1.54d) was used to perform a semiquantitative analysis of the laser confocal images. The following primary antibodies were used: rabbit polyclonal anti‐myosin 7a (1:1000; Proteus Biosciences; 25–6790), rabbit polyclonal anti‐Nrf2 (1:200; GeneTex; GTX103322), and mouse monoclonal anti‐Nox2 (1:200; Santa Cruz; sc‐74514). The following secondary antibodies were used: Alexa‐Fluor‐594‐conjugated anti‐rabbit (1:200;Invitrogen‐Thermo Fisher; A21207), Alexa‐Fluor‐488‐conjugated anti‐rabbit(1:200;Invitrogen‐Thermo Fisher; A21206), Alexa‐Fluor‐594‐conjugated anti‐mouse(1:200;Invitrogen‐Thermo Fisher; A21203).

### Dihydroethidium (DHE)

DHE (GlpBio; GC30025) was used to assess the level of intracellular superoxide anions. HBSS was preheated to 37 °C, and the culture medium in the culture dish was removed. DHE working solution (final concentration of 10 µm) prepared with Hank's solution was added, and the cells were cultured in a cell incubator at 37 °C for 30 min. Then, the cells were fixed with 4% paraformaldehyde for 20 min, costained with an anti‐myosin 7a antibody and DAPI, and observed under a laser confocal microscope.

### MitoSOX Red Staining

MitoSOX Red (Invitrogen; M36008) was used to specifically assess superoxide levels in mitochondria. In accordance with the manufacturer's instructions, after the basilar membrane culture was completed, 5 µm MitoSOX Red working solution was added, and the cells were cultured in an incubator at 37 °C for 20 min, fixed with 4% paraformaldehyde for 20 min, costained with an anti‐myosin 7a antibody and DAPI, and observed under a laser confocal microscope.

### TUNEL Staining

A TUNEL kit (Cell Death Detection Kit, 4AF488 TUNEL Assay; FXP142‐050) was used to assess the level of cell apoptosis. Cells were fixed with 4% paraformaldehyde for 20 min and treated with 0.2% Triton X‐100 for 20 min. Then, the TUNEL reaction mixture was added, and the cells were incubated at 37 °C for 2 h. Costaining with an anti‐myosin 7a antibody and DAPI was performed, and the cells were observed under a laser confocal microscope.

### Western Blotting

The cochlear tissue was placed in RIPA lysis buffer (Glpbio; GK10023) containing PMSF (1:100; Glpbio; GC10477) and a protease inhibitor cocktail (1:100; Glpbio; GK10014). The protein concentration was measured using a BCA protein assay kit (Glpbio; GK10009). A total of 30 µg of each protein sample was separated on 4–12% SDS–PAGE gels. After electrophoresis, the proteins were transferred to a polyvinylidene difluoride membrane. The membrane was incubated with a primary antibody overnight at 4 °C and then with a secondary antibody at 37 °C for 1 h. After three washes, the bands were visualized via a chemiluminescence substrate (Millipore, WBKLS0500) and analyzed using ImageJ software. The following primary antibodies were used: rabbit polyclonal anti‐Nrf2 (1:200; GeneTex; GTX103322) and mouse monoclonal anti‐β‐Actin (1:1000; Abcam; ab8226). HRP‐conjugated AffiniPure goat anti‐rabbit (or anti‐mouse) IgG (1:5000, Proteintech, SA00001‐2, SA00001‐1) was used as the secondary antibody. Each experiment was repeated more than 3 times.

### Virtual Screening of Drugs

First, the 3D structure of human NOX2 (PDB ID: 8GZ3) was downloaded from the RCSB PDB website. The Protein Preparation Wizard module was used to hydrogenate the protein, delete water molecules, delete the A/N chains and other small molecules, retain the B chain and ligand FAD, add missing chain atoms, and then perform energy optimization (OPLS2005 Force Field, RMSD 0.30 Å). Finally, the processed Nox2 protein was subjected to molecular docking in the MCE database using the Glide module; that is, the receptor and ligand molecules were docked with each other through geometric matching and energy matching, thereby screening small molecule compounds with strong binding ability to the Nox2 protein.

### Statistical Analysis

SPSS 27.0 software and GraphPad Prism 8 were used for statistical analysis, and the results are expressed as the mean ± standard deviation ($\bar{x}$±S). Baseline audiometry of ABR was performed before noise exposure, and one animal was excluded from the audiometric testing experiment because of abnormal baseline ABR. All the experiments were repeated three or more times. For in vivo experiments with mice, *n* indicates the number of mice. For cochlear explant culture experiments, *n* indicates the number of individual cochlear basilar membranes. The sample size *n* for each statistical analysis is indicated in each figure. One‐way analysis of variance and Dunnett's multiple comparison tests were used to compare the data among groups, whereas a paired sample t‐test was used to analyze differences within the same group. When α = 0.05 and *p* < 0.05, the difference was considered statistically significant.

## Conflict of Interest

The authors declare no conflict of interest.

## Author Contributions

M.H.Q., Z.J.G., and Y.Q. contributed equally to the work and performed the laboratory experiments. D.J.Z. and J.H.Q. designed this study. R.F.W., K.Y.T., and B.Y. contributed to the mouse audiology tests. M.H.Q., Z.J.G., Y.Q., Y.H., and Q.Z.W. contributed to the data analysis. Y.X.Z., P.Z, and Z.Z.L. contributed to the animal support for the study. Q.W. Z., Z.Y.M. and X.Y.Z. suggested changes to the figures. M.H.Q., Z.J.G. and J.C., D.J.Z. and J.H.Q. contributed to critical discussions. M.H.Q., Z.J.G. and Y.Q. wrote the paper. All the authors read and approved the final manuscript.

## Supporting information



Supporting Information

Supplemental Table

## Data Availability

The RNA‐seq data are available in the GEO database under accession number GSE111302.
