# Supplementary material for: NOX2 Contributes to High‐Frequency Outer Hair Cell Vulnerability in the Cochlea
Source: Adv Sci (Weinh). 2025 Jun 26;12(34):e08830. doi: 10.1002/advs.202408830 (PMC12442645; doi:10.1002/advs.202408830)
Supplement: Supplementary file 1 — Supporting Information [file ADVS-12-e08830-s001.pdf]

ADVANCED SCIENCE
Open Access

Supporting Information

for *Adv. Sci.*, DOI 10.1002/advs.202408830

NOX2 Contributes to High-Frequency Outer Hair Cell Vulnerability in the Cochlea

Meihao Qi, Zejun Gao, Yang Qiu, Renfeng Wang, Keyong Tian, Bo Yue, Xinyu Zhang, Peng Zhang, Ziqi Wu, Qingwen Zhu, Zhenzhen Liu, Zhuoyao Ma, Xueying Zhou, Yu Han, Jun Chen, Jianhua Qiu and DingJun Zha**

Supplementary Table 1.

Length, diameter and yield of OHC in the apical and basal turns of SD rats

Turn	Length（μm）	Diameter(μm)	Quantity
Apex	25.0±1.61	8.66±0.91	83±10.12
Base	20.4±1.90	8.42±0.75	28±3.13

Supplementary Table 1. The number of OHCs that could be isolated from the apical turn was significantly more than that from the basal turn. Data are presented as the mean ± standard deviation. (n = 5).

Supplementary Figure 1

The expression of Nox2 in cochlear OHCs of WT mice

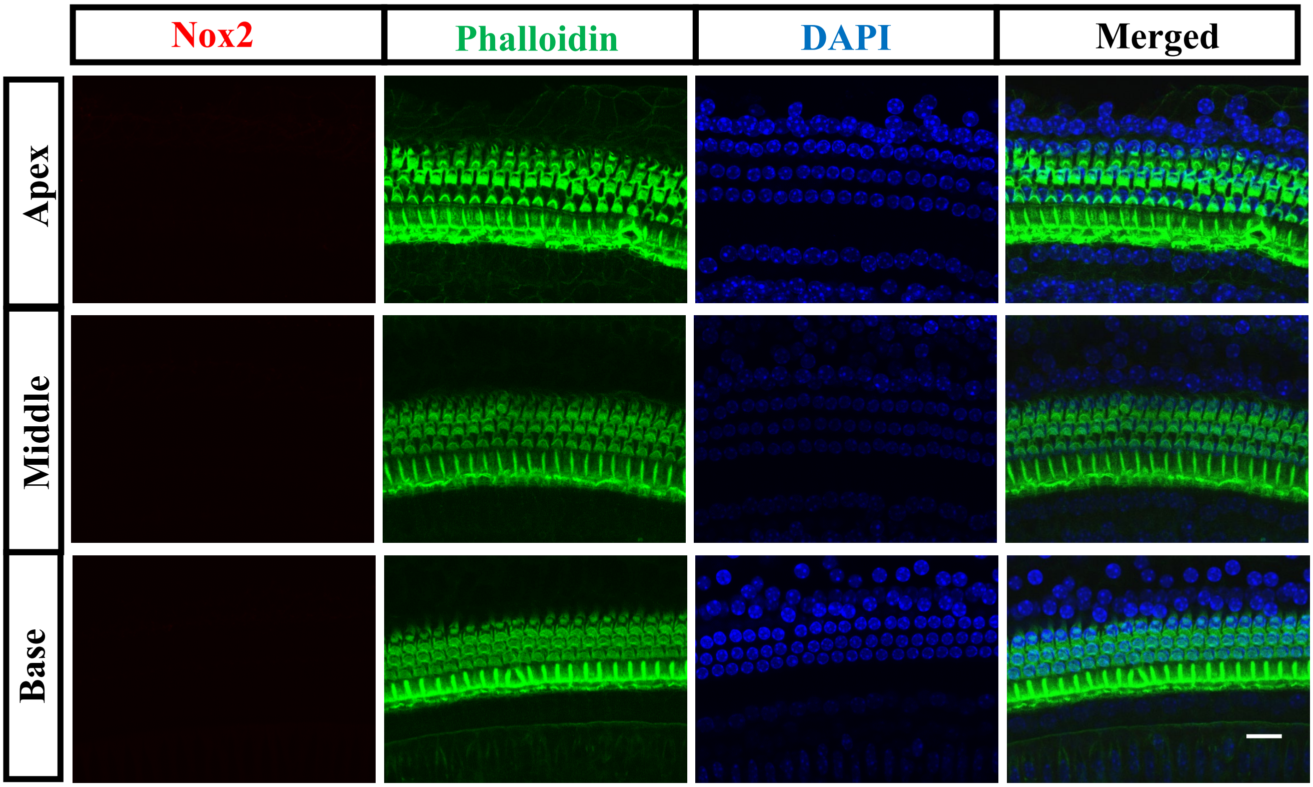

Supplementary Figure 1. Before exposure to noise or ototoxic drugs, Nox2 (red) was expressed at a low level in the OHCs of P30 C57 mice, with no difference between apical and basal OHC. Phalloidin (green) was used to label hair cells. *n* = 5. Bar= 20 μm.

Supplementary Figure 2

Identification of NOX2$^{-/-}$ mice

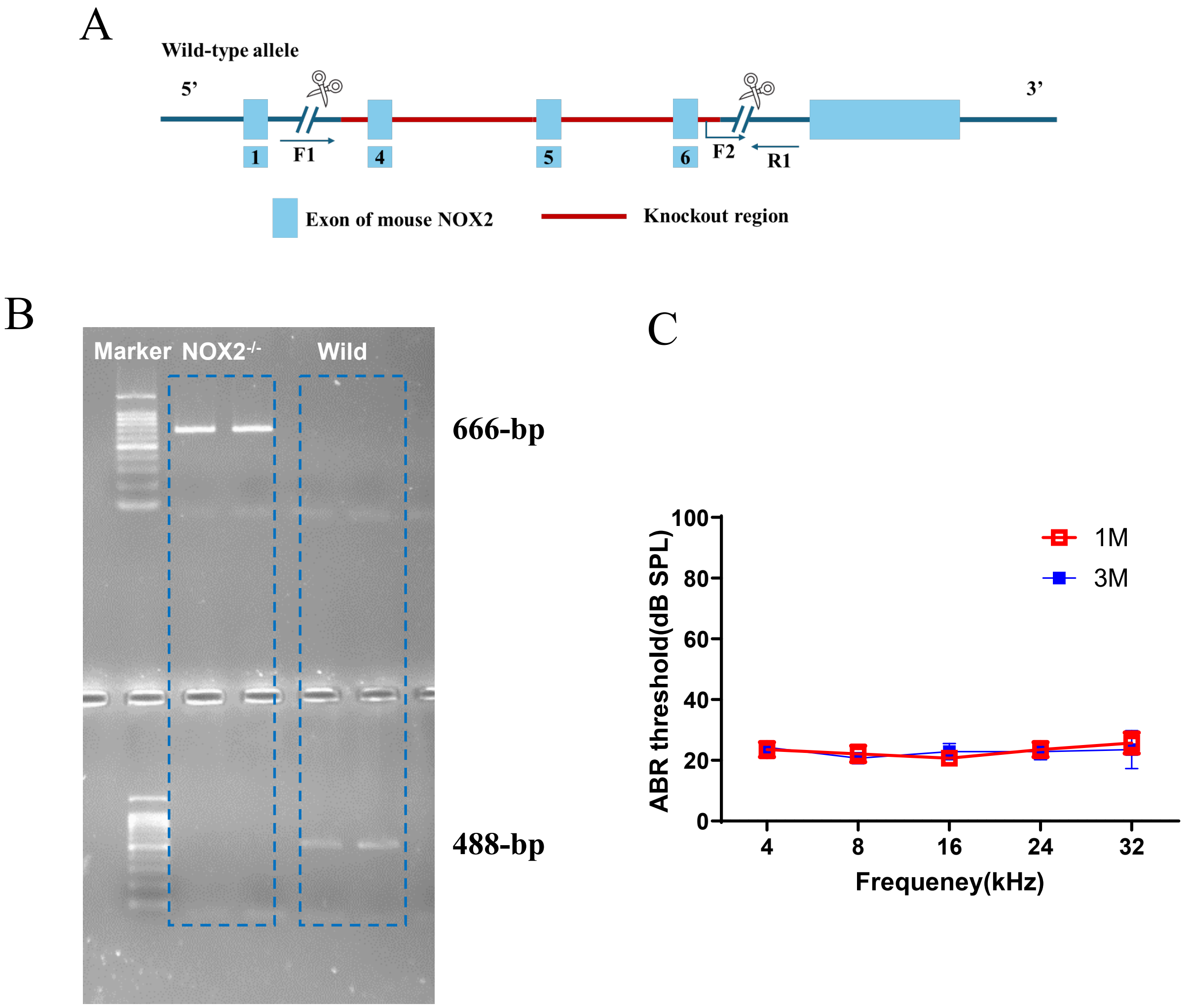

Supplementary Figure 2. Generation of C57BL/6 NOX2 knockout mice. (A) The murine NOX2 gene contains 13 exons, and the exons 4-6 were knockout. (B) PCR analysis for WT (lanes 3–4), and NOX2$^{-/-}$ (lanes 1 and 2) mice. For primer F2R1, an expected band of 488-bp was detected in WT mice. For primer F1R1, the 666-bp bands were detected in the NOX2$^{-/-}$ mice. The mice were genotyped via polymerase chain reaction (PCR) using the following primers:F1: 5'-ACTGAGAAACAATGGGGCAGATAC-3';R1: 5'-TAGCACCTGACATAACGCTTGGTAG-3';
F2: 5'-TGGTATTTGCTTTCTTGCTGTACC-3.(C) ABR thresholds of 1-month-old and 3-month-old NOX2$^{-/-}$ mice.

Supplementary Figure 3

NOX2 knockout significantly protected mice from hearing loss and hair cell damage caused by noise or neomycin

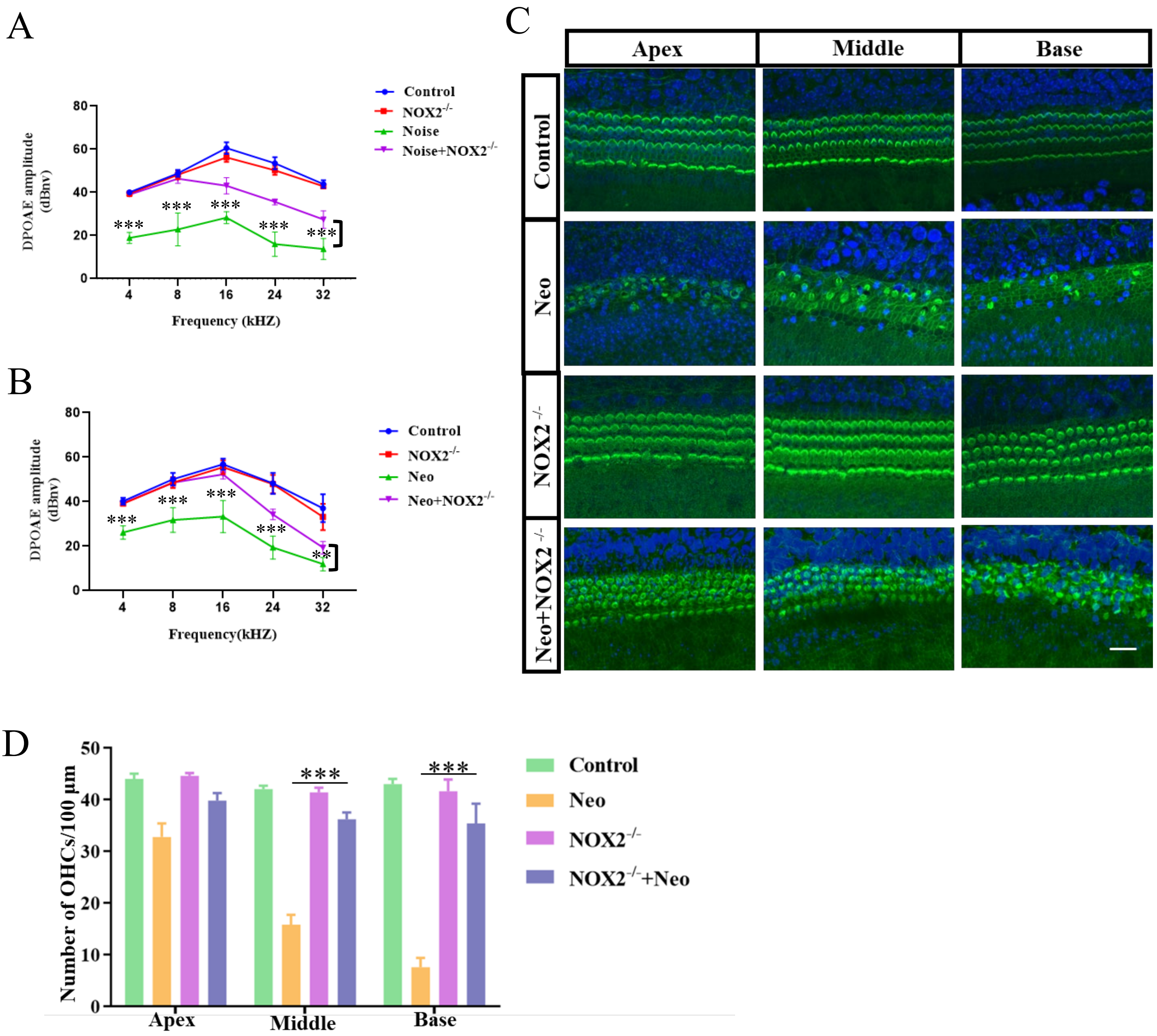

Supplementary Figure 3. NOX2 knockout significantly protected mice from hearing loss and hair cell damage caused by noise or neomycin. A) DPOAE amplitudes of four groups of mice (control group, noise group, $NOX2^{-/-}$ group and $NOX2^{-/-}$+ noise group) at frequencies of 4, 8, 16, 24 and 32kHz .14 days after noise exposure, the average DPOAE amplitude at each frequency in the noise group was significantly lower than the amplitude in the $NOX2^{-/-}$+noise group (n = 6). B) DPOAEs amplitude of the four groups of mice (control, neomycin, $NOX2^{-/-}$ and $NOX2^{-/-}$+ neomycin) at frequencies 4, 8, 16, 24 and 32kHz. Mean DPOAE amplitudes of the neomycin group were lower than amplitudes of the $NOX2^{-/-}$+neomycin group (n = 6). C)Immunofluorescence staining of the cochlear basilar membrane of mice in different treatment groups (phalloidin, green). Compared with that in the neomycin treatment group, there was a significant reduction in the loss of OHCs in mice in the $NOX2^{-/-}$ + neomycin group. D) Quantification of panel A (n = 5). Bar= 20 μm. For all experiments, * $P < 0.05$, * * $P < 0.01$, and * * * $P < 0.001$.

Supplementary Figure 4.

NOX2 knockout can inhibit neomycin-induced apoptotic pathway activation in vivo

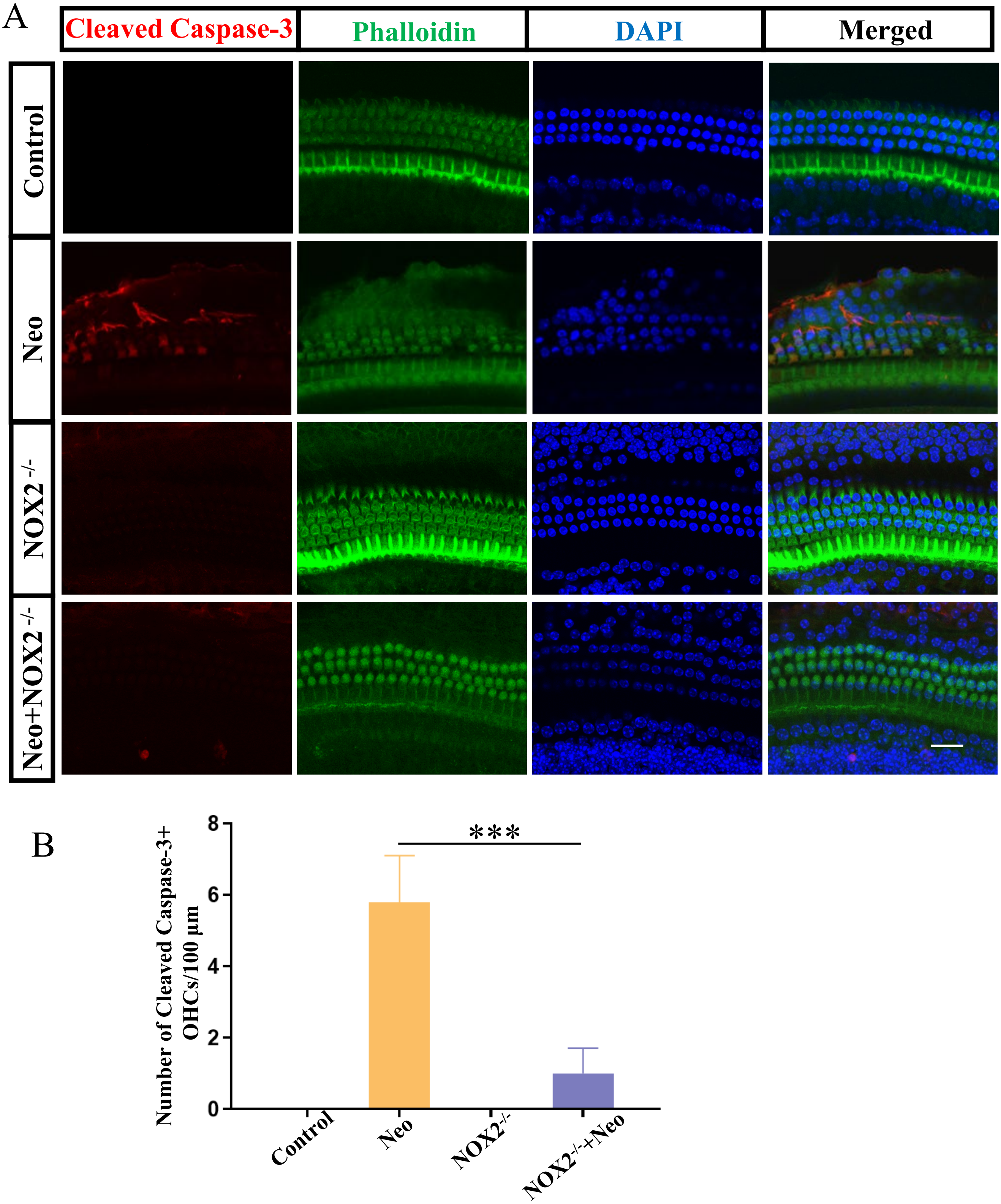

Supplementary Figure 4. NOX2 knockout can inhibit neomycin-induced apoptotic pathway activation in vivo. A) Immunofluorescence images of cleaved caspase-3 (red) expression levels in outer hair cells of mice in different treatment groups (middle turn; phalloidin, green). NOX2 knockout significantly reduced the neomycin-induced increase in the number of apoptotic outer hair cells. B) Quantitative analysis of panel A. (n = 5). Bar= 20 μm. For all experiments, * $P < 0.05$, * * $P < 0.01$, and * * * $P < 0.001$. Data are presented as the mean ± SD.
